# Gene selection for cancer classification with the help of bees

**DOI:** 10.1186/s12920-016-0204-7

**Published:** 2016-08-10

**Authors:** Johra Muhammad Moosa, Rameen Shakur, Mohammad Kaykobad, Mohammad Sohel Rahman

**Affiliations:** 1 A ℓEDA Group, Department of CSE, BUET, Dhaka-1205, Dhaka, Bangladesh; 2 Wellcome Trust - Medical Research Council Cambridge Stem Cell Institute, University of Cambridge, Cambridge, UK

**Keywords:** Gene selection, Microarray, Cancer classification, Artificial bee colony algorithm, Evolutionary algorithm

## Abstract

**Background:**

Development of biologically relevant models from gene expression data notably, microarray data has become a topic of great interest in the field of bioinformatics and clinical genetics and oncology. Only a small number of gene expression data compared to the total number of genes explored possess a significant correlation with a certain phenotype. Gene selection enables researchers to obtain substantial insight into the genetic nature of the disease and the mechanisms responsible for it. Besides improvement of the performance of cancer classification, it can also cut down the time and cost of medical diagnoses.

**Methods:**

This study presents a modified Artificial Bee Colony Algorithm (ABC) to select minimum number of genes that are deemed to be significant for cancer along with improvement of predictive accuracy. The search equation of ABC is believed to be good at exploration but poor at exploitation. To overcome this limitation we have modified the ABC algorithm by incorporating the concept of pheromones which is one of the major components of Ant Colony Optimization (ACO) algorithm and a new operation in which successive bees communicate to share their findings.

**Results:**

The proposed algorithm is evaluated using a suite of ten publicly available datasets after the parameters are tuned scientifically with one of the datasets. Obtained results are compared to other works that used the same datasets. The performance of the proposed method is proved to be superior.

**Conclusion:**

The method presented in this paper can provide subset of genes leading to more accurate classification results while the number of selected genes is smaller. Additionally, the proposed modified Artificial Bee Colony Algorithm could conceivably be applied to problems in other areas as well.

**Electronic supplementary material:**

The online version of this article (doi:10.1186/s12920-016-0204-7) contains supplementary material, which is available to authorized users.

## Background

Gene expression studies have paved the way for a more comprehensive understanding of the transcriptional dynamics afflicted on a cell under different biological stresses [1–4]. The application of microarrays as a robust and amenable system to record transcriptional profiles across a range of differing species has been growing exponentially. In particular, the evaluation of human expression profiles in both health and in disease has implications for the development of clinical bio-markers for diagnosis as well as prognosis. Hence, diagnostic models from gene expression data provide more accurate, resource efficient, and repeatable diagnosis than the traditional histopathology [5]. Indeed, microarray data is now being used in clinical applications as it is possible to predict the treatment of human diseases by analyzing gene expression data [2, 6–9]. However, one of the inherent issues with gene expression profiles are their characteristically high-dimensional noise, contributing to possible high false positive rates. This is further compounded during analysis of such data whereby the use of all genes may potentially hinder the classifier performance by masking the contribution of the relevant genes [10–15]. This has led to a critical need for the development of analytical tools and methodologies which are able to select a small subset of genes both from a practical and qualitative perspective. As a result the selection of discriminatory genes is essential to improve the accuracy and also to decrease the computational time and cost [16].

The classification of gene expression samples involves feature selection and classifier design. However, noisy, irrelevant, and misleading attributes make the classification task complicated, given they can contain erroneous correlations. A reliable selection method of relevant genes for sample classification is needed in order to increase classification accuracy and to avoid incomprehensibility. The task of gene selection is known as feature selection in artificial intelligence domain. Feature selection has class-labeled data and attempts to determine which features best distinguish among the classes. The genes are considered to be the features that describe the cell. The goal is to select a minimum subset of features that achieves maximum classification performance and to discard the features having little or no effect. These selected features can then be used to classify unknown data. Feature selection can thus be considered as a principle pre-processing tool when solving classification problems [17, 18]. Theoretically, feature selection problems are NP-hard. Performing an extensive search is impossible as the computational time and cost would be excessive [19].

Gene selection methods can be divided into two categories [20]: filter methods, and wrapper or hybrid methods. Detail review on gene selection methods can be found in [20–25]. A gene selection method is categorized as a filter method if it is carried out independently from a classification procedure. In filter approach instead of searching the feature space, selection is done based on statistical properties. Due to lower computational time and cost most previous gene selection techniques in the early era of microarrays analysis have used the filter method. Many filters provide a feature ranking rather than an explicit best feature subset. The top ranking features are chosen manually or via cross-validation [26–28] while the remaining low ranking features are eliminated. Bayesian Network [29], *t*-test [30], Information Gain (IG) and Signal-to-Noise-Ratio (SNR) [5, 31], Euclidean Distance [32, 33], etc. are the examples of filter methods that are usually considered as individual gene-ranking methods. Filter methods generally rely on a relevance measure to assess the importance of genes from the data, ignoring the effects of the selected feature subset on the performance of the classifier. This may however result in the inclusion of irrelevant and noisy genes in a gene subset. Research shows that, rather than acting independently, genes in a cell interact with one another to complete certain biological processes or to implement certain molecular functions [34].

While the filter methods handle the identification of genes independently, a wrapper or hybrid method on the other hand, implements a gene selection method merging with a classification algorithm. In the wrapper methods [35] a search is conducted in the space of genes, evaluating the fitness of each found gene subset. Fitness is determined by training the specific classifier to be used only with the found gene subset and then approximating the accuracy percentage of the classifier. The hybrid methods usually obtain better predictive accuracy estimation than the filter methods [36–39], since the genes are selected by considering and optimizing the correlations among genes. Therefore, several hybrid methods have been implemented to select informative genes for binary and multi-class cancer classification in recent times [37, 40–50]. However, its computational cost must be taken into account [39]. Notably, filter methods have also been used as a preprocessing step for wrapper methods, allowing a wrapper to be used on larger problem instances.

Recently many diverse population based methods have been developed for investigating gene expression data to select a small subset of informative genes from the data for cancer classification. Over the time a number of variants and hybrids of Particle Swarm Optimization (PSO) have been proposed to solve the gene selection problem. The Combat Genetic Algorithm (CGA) [51, 52] has been embedded within the Binary Particle Swarm Optimization (BPSO) in [44] which serves as a local optimizer at each iteration to improve the solutions of the BPSO. The algorithm has succeeded to achieve high classification accuracy albeit at the cost of unacceptably large size of the selected gene set. Although both PSO and CGA perform well as global optimizer, the proposed algorithm has failed to obtain satisfactory results because of not considering minimization of selected gene size as an objective. Also Li et al. [41] presented a hybridization of BPSO and Genetic Algorithm (GA). However, its performance is not satisfactory enough. Shen et al. [40] discussed incorporation of Tabu Search (TS) in PSO as a local improvement procedure to maintain the population diversity and prevent steering to misleading local optima. Obtained accuracy by their hybrid algorithm is sufficient but they did not provide any discussion at all about the number of genes selected. Again BPSO has been embedded in TS by Chuang et al. [42] to prevent TS form getting trapped in local optima which helps in achieving satisfactory accuracy for some of the datasets. However, to attain that accuracy their algorithm needs to select prohibitively high number of genes. An improved binary particle swarm optimization (IBPSO) is proposed by Chuang et al. [43] which achieves good accuracy for some of the datasets but, again, selects high number of genes. Recently, Mohamad et al. [37] have claimed to enhance the original BPSO algorithm by minimizing the probability of gene to be selected, resulting in the selection of only the most informative genes. They have obtained good classification accuracy with low number of selected genes for some of the datasets. But the number of iterations to achieve the target accuracy is higher than ours, which will be reported in the Results and discussion Section of this paper. A simple modified ant colony optimization (ACO) algorithm is proposed by Yu et al. in [53], which associates two pheromone components with each gene rather than a single one as follows. One component determines the effect of selecting the gene whether the other determines the effect of not selecting it. The algorithm is evaluated using five datasets. It is able to select small number of genes and accuracy is also reasonable. Random forest algorithm for classifying microarray data [54] has obtained good accuracy for some datasets but not for all. Notably, the number of selected genes by the random forest classification algorithm in [54] has been found to be high for some of the datasets. A new variable importance measure based on the difference of proximity matrix has been proposed for gene selection using random forest classification by Zhou et al. [55]. Although it fails to achieve the highest accuracy for any dataset, their algorithm is able to select small number of genes and achieve satisfactory accuracy for all the datasets. Recently, Debnath and Kurita [56] have proposed an evolutionary SVM classifier that adds features in each generation according to the error-bound values for the SVM classifier and frequency of occurrence of the gene features to produce a subset of potentially informative genes.

In this paper, we propose a modified artificial bee colony algorithm to select genes for cancer classification. The Artificial Bee Colony (ABC) algorithm [57], proposed by Karaboga in 2005, is one of the most recent swarm intelligence based optimization technique, which simulates the foraging behavior of a honey bee swarm. The search equation of ABC is reported to be good at exploration but poor at exploitation [58, 59]. To overcome this limitation we have modified the ABC algorithm by incorporating the concept of pheromone which is one of the major components of the Ant Colony Optimization (ACO) algorithm [60, 61] and a new operation in which successive bees communicate to share their results. Even though researchers are unable to establish whether such a communication indeed involve information transfer or not, it is known that foraging decisions of outgoing workers, and the probability to find a recently-discovered food source, are influenced by the interactions [62–67]. Indeed, there is a notable proof that for harvester ants, management of foraging activity is guided by ant encounters [68–71]. Even the mere instance of an encounter may provide some information, such as, the magnitude of the colony’s foraging activity, and may therefore influence the probability of food collection by ants [72–74].

We believe that the selection of genes by our system provide us some interesting clue towards the importance and contribution of that set of particular genes for the respective cancer disease. To elaborate, our system has identified that for diffuse large B-cell lymphoma (DLBCL) only three (3) genes are informative enough to decide about the cancer. Now, this could turn out to be a string statement with regards to the set of genes identified for a particular cancer and we believe further biological validation is required before making such a string claim. We do plan to work towards validation of these inferences.

During the last decade, several algorithms have been developed depending on different intelligent behaviors of honey bee swarms [57, 75–85]. Among those, ABC is the one which has been most widely studied on and applied to solve the real world problems, so far. Comprehensive study on ABC and other bee swarm algorithms can be found in [86–89]. The algorithm has the advantage of sheer simplicity, high flexibility, fast convergence, and strong robustness which can be can be used for solving multidimensional and multimodal optimization problems [90–92]. Since the ABC algorithm was proposed in 2005, it has been applied in many research fields, such as flow shop scheduling problem [93, 94], parallel machines scheduling problem [95], knapsack problem [96], traveling salesman problem [97], quadratic minimum spanning tree problem [98], multiobjective optimization [99, 100], generalized assignment problem [101], neural network training [102], and synthesis [103], data clustering [104], image processing [105], MR brain image classification [106], coupled ladder network [107], wireless sensor network [108], vehicle routing [109], nurse rostering [110], computer intrusion detection [111], live virtual machine migration [112], etc. Studies [86, 113] have indicated that ABC algorithms have high search capability to find good solutions efficiently. Besides, excellent performances has been reported by ABC for a considerable number of problems [98, 100, 114]. Karaboga and Basturk [113] tested for five multidimensional numerical benchmark functions and compared the ABC performance with that of Differential Evolution (DE), Particle Swarm Optimization (PSO) and Evolutionary Algorithm (EA). The study concluded that ABC gets out of a local minimum more efficiently for multivariable and multimodal function optimization and outperformed DE, PSO and EA.

However, it has been observed that the ABC may occasionally stop proceeding toward the global optimum even though the population has not converged to a local optimum [86]. Research [58, 59, 115] shows that the solution search equation of ABC algorithm is good at exploration but unsatisfactory at exploitation. For the population based algorithms the exploration and the exploitation abilities are both necessary features. The exploration ability refers to the ability to investigate the various unknown regions to discover the global optimum in solution space, while the exploitation ability refers to the ability to apply the knowledge of the previous good solutions to find better solutions. The exploration ability and the exploitation ability contradict to each other, so that the two abilities should be well balanced to achieve good performance on optimization problems. As a result, several improvements of ABC have been proposed over the time. Baykasoglu et al. [101] incorporated the ABC algorithm with shift neighborhood searches and greedy randomized adaptive search heuristic and applied it to the generalized assignment problem. Pan et al. [93] proposed a Discrete Artificial Bee Colony (DABC) algorithm with a variant of iterated greedy algorithm with total weighted earliness and tardiness penalties criterion. Li et al. [116] used a hybrid Pareto-based ABC algorithm to solve flexible job shop-scheduling problems. In the proposed algorithm, each food sources is represented by two vectors, the machine assignment and the operation scheduling. Wu et al. [117] combined Harmony Search (HS) and the ABC algorithm to construct a hybrid algorithm. Comparison results show that the hybrid algorithm outperforms ABC, HS, and other heuristic algorithms. Kang et al. [118] anticipated a Hooke Jeeves Artificial Bee Colony algorithm (HJABC) for numerical optimization. HJABC integrates a new local search named ‘modus operandi’ which is based on Hooke Jeeves method (HJ) [119] with the basic ABC. Opposition Based Lévy Flight ABC is developed by Sharma et al. [120]. Lévy flight based random walk local search is proposed and incorporated with ABC to find the global optima. Szeto et al. [109] proposed an enhanced ABC algorithm. The performance of the new approach is tested on two sets of standard benchmark instances. Simulation results show that the newly proposed algorithm outperforms the original ABC and several other existing algorithms. Chaotic Search ABC (CABC) is introduced by Yan et al. [121] to solve the premature convergence issue of ABC by increasing the number of scout and rational using of the global optimal value and chaotic Search. Again a Scaled Chaotic ABC (SCABC) method is proposed in [106] based on fitness scaling strategy and chaotic theory. Based on the Rossler attractor of chaotic theory a novel Chaotic Artificial Bee Colony (CABC) is developed in [122] to improve the performance of ABC. An Improved Artificial Bee Colony (IABC) algorithm is proposed in [123] to improve the optimization ability of ABC. The paper introduces an improved solution search equation in employee and scout bee phase using the best and the worst individual of the population. In addition, the initial population is generated by the piecewise logistic equation which employs chaotic systems to enhance the global convergence. Inspired by Differential Evolution (DE), Gao et al. [124] proposed an improved solution search equation. In order to balance the exploration of the solution search equation of ABC and the exploitation of the proposed solution search equation, a selective probability is introduced. In addition, to enhance the global convergence, when producing the initial population, both chaotic systems and opposition based learning methods are employed. Kang et al. [91] proposed a Rosenbrock ABC (RABC) algorithm which combines Rosenbrock’s rotational direction method with the original ABC. There are two alternative phases of RABC: the exploration phase realized by ABC and the exploitation phased completed by the Rosenbrock method. Tsai et al. [125] introduced the Newtonian law of universal gravitation in the onlooker phase of the basic ABC algorithm in which onlookers are selected based on a roulette wheel to maximize the exploitation capacity of the solutions in this phase and the strategy is named as Interactive ABC (IABC). The IABC introduced the concept of universal gravitation into the consideration of the affection between employed bees and the onlooker bees. The onlooker bee phase is altered by biasing the direction towards random bee according to its fitness. Zhu and Kwong [115] utilized the search information of the global best solution to guide the search of ABC to improve the exploitation capacity. The main idea is to apply the knowledge of the previous good solutions to find better solutions. Reported results show that the new approach achieves better results than the original ABC algorithm. Banharnsakun et al. [126] modified the search pattern of the onlooker bees such that the solution direction is biased toward the best-so-far position. Therefore, the new candidate solutions are similar to the current best solution. Li et al. [58] proposed an improved ABC algorithm called I-ABC, in which the best-so-far solution, inertia weight, and acceleration coefficients are introduced to modify the search process. The proposed method is claimed to have an extremely fast convergence speed. Gbest guided position update equations are introduced in Expedited Artificial Bee Colony (EABC) [127]. Jadon et al. [128] proposed an improved ABC named as ABC with Global and Local Neighborhoods (ABCGLN) which concentrates to set a trade off between the exploration and exploitation and therefore increases the convergence rate of ABC. In the proposed strategy, a new position update equation for employed bees is introduced where each employed bee gets updated using best solutions in its local and global neighborhoods as well as random members from these neighborhoods. With a motivation to balance exploration and exploitation capabilities of ABC, Bansal et al. [129] presents an self adaptive version of ABC named as SAABC. In this adaptive version, to give more time to potential solutions to improve themselves, the parameter ‘*limit*’, of ABC is modified self adaptively based on current fitness values of the solutions. This setting of ‘*limit*’ makes low fit solutions less stable, which helps in exploration. Also to enhance the exploration, number of scout bees are increased. To achieve an improved ABC based approach with better global exploration and local exploitation ability, a novel heuristic approach named PS-ABC is introduced by Xu et al. [112]. The method utilizes the binary search idea and the Boltzmann selection policy to achieve the uniform random initialization and thus to make the whole PSABC approach have a better global search potential and capacity at the very beginning. To obtain more efficient food positions Sharma et al. [130] introduced two new mechanisms for the movements of scout bees. In the first method, the scout bee follows a non-linear (quadratic) interpolated path while in the second one, scout bee follows Gaussian movement. The first variant is named as QABC, while the second variant is named as GABC. Numerical results and statistical analysis of benchmark unconstrained, constrained and real life engineering design problems indicate that the proposed modifications enhance the performance of ABC. In order to improve exploitation capability of ABC a new search pattern is proposed by Xu et al. [131] for both employed and onlooker bees. In the new approach, some best solutions are utilized to accelerate the convergence speed. A solution pool is constructed by storing some best solutions of the current swarm. New candidate solutions are generated by searching the neighborhood of solutions randomly chosen from the solution pool. Kumar et al. [97] added crossover operators to the ABC as the operators have a better exploration property. Ji et al. [96] developed a new ABC algorithm combining chemical communication way and behavior communication way based on researches of entomologists. The new ABC algorithm introduces a novel communication mechanism among bees. In order to have a better coverage and a faster convergence speed, a modified ABC algorithm introducing forgetting and neighbor factor (FNF) in the onlooker bee phase and backward learning in the scout bee phase is proposed by Yu et al. [108]. Bansal et al. [132] introduced Memetic ABC (MeABC) in order to balance between diversity and convergence capability of the ABC. A new local search phase is integrated with the basic ABC to exploit the search space identified by the best individual in the swarm. In the proposed phase, ABC works as a local search algorithm in which, the step size that is required to update the best solution, is controlled by Golden Section Search (GSS) [133] approach. In the memetic search phase new solutions are generated in the neighborhood of the best solution depending upon a newly introduced parameter, perturbation rate. Kumar et al. [134] also proposed memetic search strategy to be used in place of employed bee and onlooker bee phase. Crossover operator is applied to two randomly selected parents from current swarm. After crossover operation two new offspring are generated. The worst parent is replaced by the best offspring, other parent remains same. The experimental result shows that the proposed algorithm performs better than the basic ABC without crossover in terms of efficiency and accuracy. Improved onlooker bee phase with help of a local search strategy inspired by memetic algorithm to balance the diversity and convergence capability of the ABC is proposed by Kumar et al. [135]. The proposed algorithm is named as Improved Onlooker Bee Phase in ABC (IOABC). The onlooker bee phase is improved by introducing modified GSS [133] process. Proposed algorithm modifies search range of GSS process and solution update equation in order to balance intensification and diversification of local search space. Rodriguez et al. [95] combined two significant elements with the basic scheme. Firstly, after producing neighboring food sources (in both the employed and onlooker bees phases), a local search is applied with a predefined probability to further improve the quality of the solutions. Secondly, a new neighborhood operator based on the iterated greedy constructive-destructive procedure [136, 137] is proposed. For further discussion please refer to the available reviews on ABC [138]. Several algorithms have been introduced that incorporates idea of ACO or PSO in bee swarm based algorithms. But our approach is unique and different from others. Hybrid Ant Bee Colony Algorithm (HABC) [139] considers pheromone for each candidate solution. On the other hand we incorporated pheromone for each gene (solution components). Our approach to find neighboring solution is different from basic ABC. But HABC follows the same neighbor production mechanism as basic ABC. In our algorithm pheromone deposition is done after each bee stage. While selecting a potential candidate solution we depend on its fitness, but HABC selects a candidate depending upon its pheromone value. Most importantly in our algorithm scout bees make use of the pheromone while exploring to find new food source. Ji et al. [96] proposed an artificial bee colony algorithm merged with pheromone. In this paper scouts are guided by pheromone along with some heuristic information while we only make use of pheromone. The paper updates pheromone only in the employed bee stage while we update pheromone in all the bee stages. Pheromone laying is done by depositing a predefined constant amount. But amount of pheromone we have deposited is a function of fitness measures. Kefayat et al. [140] proposed a hybrid of ABC and ACO. Inside loop contains the ABC and outside loop is ACO without any modification. ABC is applied in the inner loop to optimize a certain constraint (source size) for each ant. Zhu et al. [115] uses ABC in a problem with continuous space. We indirectly guide scout through the best found solutions whether this paper guides the employed and onlooker bees.

## Methods

Gene expression profiles provide a dynamic means to molecularly characterise the state of a cell and so has great potential as clinical diagnostic and prognostic tool. However, in comparison to the number of genes involved which often exceeds several thousands, available training datasets generally have a fairly small sample size for classification. Hence, inclusion of redundant genes decreases the quality of classification thus increasing false positive rates. To overcome this problem one of the approaches in practice is to search for the informative genes along with applying a filter beforehand. Use of confidently pre-filtering makes it possible to get rid of the majority of redundant noisy genes. Consequently, the underlying method to search the informative genes becomes easier and efficient with respect to time and cost. Finally, to evaluate the fitness of the selected gene subset a classifier is utilized. The selected genes are used as features to classify the testing samples. The inputs and outputs of the method are:

- Input: *G*={*G*_1_,*G*_2_,…,*G*_*n*_}, a vector of vectors, where *n* is the number of genes and *G*_*i*_={*g*_*i*,1_,*g*_*i*,2_,…,*g*_*i,N*_} is a vector of gene expressions for the *i*^*th*^ gene where *N* is the sample size. So, *g*_*i,j*_ is the expression level of the *i*^*th*^ gene in the *j*^*th*^ sample.
- Output: *R*={*R*_1_,*R*_2_,…,*R*_*m*_}, the indices of the genes selected in the optimal subset. Where *m* is the selected gene size.

The gene selection method starts with a preprocessing step followed by a gene selection algorithm. Finally the classification is done. In what follows, we will describe these steps in detail.

### Preprocessing

To make the experimental data suitable for our algorithm and to help the algorithm run faster the preprocessing step is incorporated. The preprocessing step contains the following two stages:

- Normalization
- Prefilter

**Normalization** Normalizing the data ensures the allocation of equal weight to each variable by the fitness measure. Without normalization, the variable with the largest scale will dominate the fitness measure [141]. Therefore, normalization reduces the training error, thereby proving the accuracy for the classification problem [142]. The expression levels for each gene are normalized at this step to [0, 1] using the standard procedure which is shown in Eq. 1 below.

1 $${} x=lower + \left[ upper - lower \times \frac{value-value\_min}{value\_max-value\_min} \right]  $$

Here, among all the expression levels of the gene in consideration, *value*_*max* is the maximum original value, *value*_*min* is the minimum original value, *upper* (*lower*) is 1 (0) and *x* is the normalized expression level. So for all gene after normalization, *value*_*max* will be 1 and *value*_*min* will be 0.

**Prefilter** Gene expression data are characteristically multi faceted given the inherent biological complexity such networks reside in. The huge number of genes causes great computational complexity in wrapper methods when searching for significant genes. Before applying other search methods it is thus prudent to reduce gene subset space by pre-selecting a smaller number of informative genes based on some filtering criteria. Several filter methods have been proposed in the literature which can be used to preprocess data. These include Signal-to-Noise Ratio (SNR) and Information Gain (IG) [5, 31], *t*-test [30], Bayesian Network [29], Kruskal-Wallis non-parametric analysis of variance (ANOVA) algorithm [143–145], *F*-test (ratio of in between group variance to within group variance) [146, 147], BW ratio [148], Euclidean Distance [32, 33], etc. After the prefilter stage, we get a ranking of the genes based on the applied statistical methods.

Because of the nature of gene expression data the selected statistical method should be able to deal with high dimensional small sample sized data. According to the assumption of the data characteristics two types of filtering methods exist, namely, parametric and non parametric. Both types of filtering techniques have been employed individually in our proposed algorithm for the sake of comparison. Among many alternatives, in our work, Kruskal Wallis [143–145] and *F*-test [146, 147] are employed individually to rank the genes. Notably, the former is a non parametric method and the latter is a parametric one.

**Kruskal Wallis (KW)** The Kruskal-Wallis rank sum test (named after William Kruskal and W. Allen Wallis) is an extension of the Mann-Whitney U or Wilcoxon Sum Rank test [149, 150] for comparing two or more independent samples that may have different sample sizes [143–145]. The Kruskal-Wallis rank sum test (KWRST) is the non-parametric equivalent to the one-way Analysis of Variance (ANOVA). It compares several populations on the basis of independent random samples from each population by determining whether the samples belong to the same distribution. Assumptions for the Kruskal-Wallis test are that within each sample, the observations are independent and identically distributed and the samples are independent of each other. It makes no assumptions about the distribution of the data (e.g., normality or equality of variance) [151, 152]. According to the results found by Deng et al. [153], the assumption about the data distribution often does not hold in gene expression data. The Kruskal-Wallis test is in fact very convenient for microarray data because it does not require strong distributional assumptions [154], it works well on small samples [155], it is suited for multiclass problems, and its *p*-values can be calculated analytically. The Kruskal-Wallis test is utilized to determine *p*-values of each gene. The genes are then sorted in increasing order of the *p*-values. The lower the *p*-value of a gene, the higher the rank of the gene. The steps of the Kruskal-Wallis test are given below:

Step 1 For each gene expression vector *G*_*i*_,
  We rank all gene expression levels across all classes. We Assign any tied values the average of the ranks they would have received if they had not been tied.
  We calculate the test statistics *K*_*i*_ for gene expression vector *G*_*i*_ of the *i*^*th*^ gene, which is given by Eq. 2 below:
    2 $$ K_{i}=\frac{12}{N(N+1)} \sum\limits_{k=1}^{C_{i}} {n^{i}_{k}} \left(\bar{r}_{k}^{i}-\frac{(N+1)}{2}\right)^{2}  $$
    Here, for the *i*^*th*^ gene,
    *N* is the sample size,
    *C*_*i*_ is the number of different classes,
    ${n^{i}_{k}}$ is the number of expression levels that are from class *k*, and
    $\bar {r}_{k}^{i}$ is the mean of the ranks of all expression level measurements for class *k*.
  If ties are found while ranking data in the *i*^*th*^ gene, correction of ties must be done. For this correction, *K*_*i*_ is divided by: $(1-\frac {\sum _{j=1}^{T_{i}} {t_{j}^{3}}-t_{j}}{ N^{3}-N })$, where *T*_*i*_ is the number of groups of different tied ranks for the *i*^*th*^ gene and *t*_*j*_ is the number of ties within group *j*.
  Finally the *p*-value for the *i*^*th*^ gene, *p*_*i*_ is approximated by $Pr(\chi ^{2}_{C_{i}-1} \geq K_{i})$, where $\chi ^{2}_{C_{i}-1}$ refers to the critical chi-square value. To compute the *p*-values, necessary functions of the already implemented package from https://svn.win.tue.nl/trac/prom/browser/Packages/Timestamps/Trunk/src/edu/northwestern/at/utils/math/are incorporated in our method.
Step 2 After the *p*-values for all the genes are calculated, we rank each gene *G*_*i*_ according to *p*_*i*_. The lower the *p*-value of a gene, the higher is its ranking.

Kruskal-Wallis is used as a preprocessing step in many gene selection algorithms [156–158]. Kruskal-Wallis test is utilized to rank and pre-select genes in the two-stage gene selection algorithm proposed by Duncan et al. [158]. In the proposed method the number of genes selected from the ranked genes is optimized by cross-validation on the training set. Wang et al. [157] applied Kruskal-Wallis rank sum test to rank all the genes for gene reduction. Obtained results from their study indicate that gene ranking with Kruskal-Wallis rank sum test is very effective. To select an initial informative subset of tumor related genes Kruskal-Wallis rank sum test is utilized by Wang et al. [156].

Besides applying Kruskal-Wallis in prefiltering stage the use of the algorithm for gene selection is also well studied [159, 160]. Chen et al. [160] studied application of different test statistics including Kruskal-Wallis for gene selection. Lan et al. [159] applied Kruskal-Wallis to rank the genes. Finally the top ranked genes are selected as features for the target task classifier. The proposed filter is claimed to be suitable as a preprocessing step for an arbitrary classification algorithm.

Like many other non-parametric tests Kruskal-Wallis uses data rank rather than raw values to calculate the statistic. However, by ranking the data some information about the magnitude of differences between scores is lost. For this reason a parametric method called *F*-test has been applied separately from Kruskal-Wallis to prefilter the genes. Notably, replacing original scores with ranks does not naturally lead to performance reduction; it rather can result in a better performance at best and a slight degradation at worst.

***F*-test** Another approach to identify the genes that are correlated to the target classes from gene expression data is by using the *F*-test [146, 147]. *F*-test is one of the most widely used supervised feature selection methods. The key idea is to find a subset of features, such that the distances between the data points in different classes are as large as possible, while the distances between the data points in the same class are as small as possible in the data space spanned by the selected features. It uses variations among *means* to estimate variations among individual measurements. *F*-score for a gene is the ratio of in between group variance to within group variance, where each class label forms a group. The steps to compute the *F*-score are given below:

- For each gene expression vector *G*_*i*_, we compute the Fisher score (i.e., *F*-Score). The fisher score for the *i*^*th*^ gene is given by Eq. 3 below.
  3 $$ F_{i}=\frac{\sum_{k=1}^{C_{i}} {n_{k}^{i}} ({\mu_{k}^{i}}- \mu^{i})^{2}}{ \sum_{k=1}^{C_{i}} {n_{k}^{i}} ({\sigma_{k}^{i}})^{2}}  $$
  Here for the *i*^*th*^ gene,
  *μ*^*i*^ is the mean for all the gene expression levels corresponding to the *i*^*th*^ gene,
  ${\mu _{k}^{i}} $ and ${\sigma _{k}^{i}}$ are mean and standard deviation of the *k*^*th*^ class respectively,
  *C*_*i*_ is the number of classes, and
  ${n_{k}^{i}}$ is the number of samples associated with the *k*^*th*^ class.
- After computing the Fisher score for each genes, genes are sorted according to the *F*- score. The higher the *F*-score of a gene, the higher is its rank.

*F*-test has been proved to be effective for determining the discriminative power of genes [161]. Use of *F*-test either as a sidekick in gene selection [158, 162] or as a stand-alone gene selection tool [163] both are practiced in the literature. Duncan et al. [158] used *F*-test as one of the ranking schemes to preselect the genes. Guo et al. [163] proposed a privacy preserving algorithm for gene selection using *F*-criterion. The proposed method can be used in other feature selection problems. Au et al. [162] implemented *F*-test as a criterion function in their proposed algorithm to solve the problem of gene selection. Cai et al. [164] pre-selected top 1,000 genes from each dataset according to Fisher’s ratio. To guide the search their method evaluated discriminative power of features independently according to Fisher criterion. Salem et al. [165] reduced the total number of genes in the input dataset to a smaller subset using *F*-score.

The *F*-score is computed independently for each gene, which may lead to a suboptimal subset of features. Generally, the *F*-test is sensitive to non-normality [166, 167]. Thus the preferred test to use with microarray data is the Kruskal-Wallis test rather than the *F*-test since the parametricity assumption of data distribution often does not hold for gene expression data [153].

**Pre-selection of genes** The top ranked genes will enter the next phase. After the genes are ranked according to the statistical method in use, we need to calculate the number of genes to nominate for the next stage. There could be two ways to determine the number of genes to be selected in this stage. Select according to *p* In this approach we predetermine a threshold and select all the genes that have statistics calculated by Kruskal-Wallis (*F*-test) below (above) the threshold. This approach generally tends to select comparatively large number of genes [157]. To determine a suitable threshold value we have conducted scientific parameter tuning in the range of [ 0,1]. The analysis is presented in the Additional file 1. Select according to *n* Another approach is to select a predetermined number of top ranked genes. The number of genes selected from the ranked genes can be either fixed or optimized by cross-validation on the training set. EPSO [37] empirically determined a fixed number (500) and used it for all the datasets. Also several other works in the literature used this approach to preselect genes [41, 53, 156, 158, 168]. Li et al. [41] selected 40 top genes with the highest scores as the crude gene subset using Wilcoxon sum rank test. Yu et al. [53] presented detail information about top 10 marker genes. Initially Wang et al. [156] selected 300 top-ranked genes by KWRST. Duncan et al. [158] considered a set of values for number of top-ranked genes. Based on Fisher’s ratio top 1000 genes are selected by Zhou et al. [168]. But the problem in this approach is that different datasets have different sizes. So a fixed value might not be optimal for all the datasets. Determining a value that is good for all the datasets is not possible. So in this article we have selected a percentage of top ranked genes. As a result number of genes selected will depend on the original size of the dataset. Therefore, when the percentage is set to 0.1, only the top 10 *%* from the ranked genes are supplied to the next stage. We have scientifically tuned the parameter in the range of [ 0,1]. The analysis is presented in the Additional file 1.

### Gene selection

After the preprocessing step only the most informative genes are left. Now they are fed to the search method to further select a smaller subset of informative genes. In this paper as the search method we have used the modified artificial bee colony (mABC) algorithm as described below.

**Artificial Bee Colony** The Artificial Bee Colony (ABC) algorithm is one of the most recent nature inspired optimization algorithms based on the intelligent foraging behavior of honey bee swarm. ABC algorithm has been proposed by Karaboga in [57] and further developed in [113]. Excellent performances have been exhibited by the ABC algorithm for a considerable number of problems [90–92, 98, 100, 114].

In the ABC algorithm, foraging honey bees are categorized into three groups, namely, employed bees, onlooker bees and scout bees. Each category of honey bees symbolizes one particular operation for generating new candidate solution. Employed bees exploit the food sources. They bring nectar from different food sources to their hive. Onlooker bees wait in the hive for the information on food sources to be shared by the employed bees and search for a food source based on that information. The employed bees whose food sources have been exhausted become scouts and their solutions are abandoned [57]. Then the scout bees search randomly for new food sources near the hive without using any experience. After the scout finds a new food source, it becomes an employed bee again. Every scout is an explorer who does not have any guidance while looking for a new food, i.e., a scout may find any kind of food sources. Therefore, sometimes a scout might accidentally discover a more rich and entirely unknown food source.

The position of a food source is a possible solution to the optimization problem and the nectar amount of the food source represents the quality of the solution. The bees act as operators over the food sources trying to find the best one among them. Artificial bees attempt to discover the food sources with high nectar amount and finally the one with the highest nectar amount. The onlookers and employed bees carry out the exploitation process in the search space and the scouts control the exploration process. The colony consists of equal number of employed bees and onlooker bees. In the basic form, the number of employed bees is equal to the number of food sources (solutions) thus each employed bee is associated with one and only one food source. For further discussion please refer to the available reviews on ABC [138]. The pseudo-code of the ABC algorithm is presented in Algorithm 1.



**Fig. 1:**
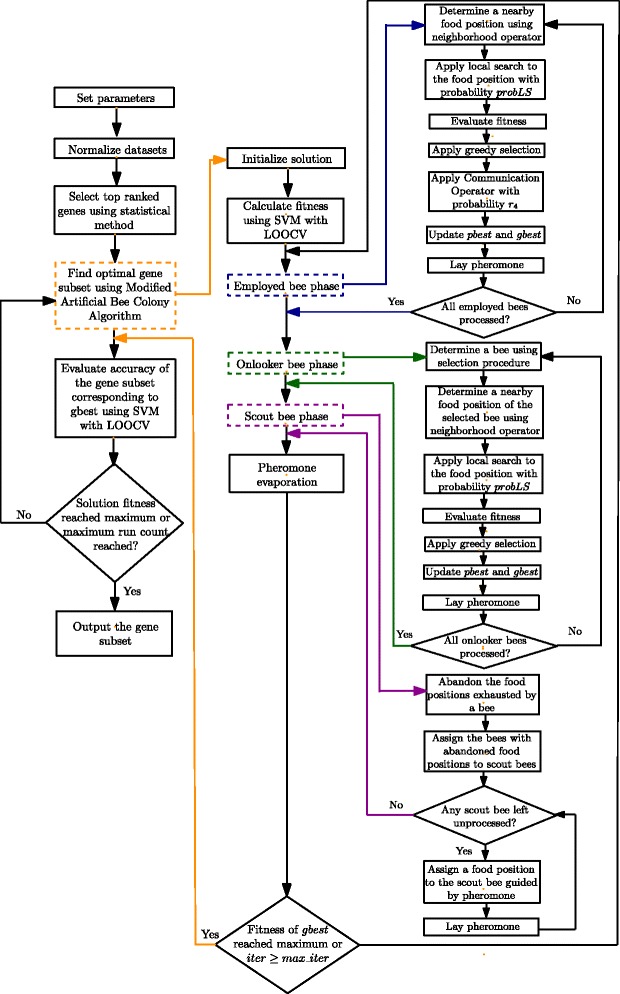
The flowchart of the modified Artificial Bee Colony (mABC) algorithm

**Modified ABC algorithm** The search equation of ABC is reported to be good at exploration but poor at exploitation [59]. As a result, several improvements of ABC have been proposed over the time [96, 97, 106, 108, 111, 112, 120–122, 124, 125, 127–132, 134]. In employed bee and onlooker bee phase, new solutions are produced by means of a neighborhood operator. In order to enhance the exploitation capability of ABC, a local search method is applied to the solution obtained by the neighborhood operator with a certain probability in [95]. To overcome the limitations of the ABC algorithm, in addition to the approach followed in [95], we have further modified it by incorporating two new components in it. Firstly, we have incorporated the concept of pheromone which is one of the major components of the Ant Colony Optimization (ACO) algorithm [60, 61]. Secondly we have introduced and plugged in a new operation named *Communication Operation* in which successive bees communicate with each other to share their results. Briefly speaking, the pheromone helps minimizing the number of selected genes while the *Communication Operation* improves the accuracy. The algorithm is iterated for *MAX*_*ITER* times. Each iteration gives a global best solution, *gbest*. Finally, the *gbest* of the last iteration, i.e., the *gbest* with maximum fitness is the output of a single run. It is worth-mentioning that finding a solution with 100 *%* accuracy is not set as the stopping criteria as further iterations can find a smaller subset with the same accuracy. Ideally, a gene subset containing only one gene with 100 *%* accuracy is the best possible solution found by any algorithm. The proposed modified ABC is given in Algorithm 11 and the flowchart can be found in Fig. 1. The modified ABC algorithm is described next.

**Food source positions** The position of the food source for the *i*^*th*^ bee *S*_*i*_, is represented by vector $X_{i}=\{x_{X_{i}}^{1}, x_{X_{i}}^{2}, \ldots, x_{X_{i}}^{n}\}$, where *n* is the gene size or dimension of the data, $x_{X_{i}}^{d} \in \{0, 1\}$, *i*=1,2,…,*m* (*m* is the population size), and *d*=1,2,…,*n*. Here, $x_{X_{i}}^{d}=1$ represents that the corresponding gene is selected, while $x_{X_{i}}^{d}=0$ means that the corresponding gene is not selected in the gene subset.

**Pheromone** We have incorporated the concept of pheromone (borrowed form ACO) to the ABC algorithm as a guide for exploitation. ACO algorithms are stochastic search procedures. The ants’ solution construction is guided by heuristic information about the problem instance being solved and (artificial) pheromone trails, which real ants use as communication media [169] to exchange information on the quality of a solution component. Pheromone helps selecting the most crucial genes. The quantity of pheromone deposited, which may depend on the quantity and quality of the food, guides other ants to the food source. Accordingly, the indirect communication via pheromone trails enables the ants to find shortest paths between their nest and food sources [169]. The gene subset carrying significant information will occur more frequently. Thus the genes in that subset will get reinforced simultaneously which ensures formation of a potential gene subset. The idea of using pheromone is to keep track of the components that are supposed to be good because they were part of a good solution in previous iterations. Because of keeping this information we need less iterations to achieve a target accuracy. Thus, computational time is also reduced.

**Pheromone update** The (artificial) pheromone trails are a kind of distributed numeric information [170] which is modified by the ants to reflect their experience accumulated while solving a particular problem. The pheromone values are updated using previously generated solutions. The update is focused to concentrate the search in regions of the search space containing high quality solutions. Solution components which are part of better solutions or are used by many ants will receive a higher amount of pheromone, and hence, will be more likely to be used by the ants in future iterations of the algorithm. It indirectly assumes that good solution components construct good solutions. However, to avoid the search getting stuck all pheromone trails are decreased by a factor before getting reinforced again. This mimics the natural phenomenon that, because of evaporation, the pheromone disappears over time unless they are revitalized by more ants. The idea of incorporating pheromone is to keep track of fitness of previous iterations.

The pheromone trails for all the components are represented by the vector *P*={*p*_1_,*p*_2_,⋯,*p*_*n*_}, where *p*_*i*_ is the pheromone corresponding to the *i*^*th*^ gene and *n* is the total number of genes. To update the pheromone *p*_*i*_ corresponding to the *i*^*th*^ gene, two steps are followed: pheromone deposition, and pheromone evaporation.

After each step of update, if the pheromone value becomes greater (less) than *tmax* (*tmin*), then the value of pheromone is set to *tmax* (*tmin*). Use of *tmax*, *tmin* is introduced in the Max-Min Ant System (MMAS) presented in [61] to avoid stagnation. The value of *tmin* is set to 0 and will be kept same throughout. But the value of *tmax* is updated whenever new global best, *gbest* solution is found. Pheromone deposition After each iteration the bees acquire new information and update their knowledge of local and global best locations. The best position found so far by the *i*^*th*^ bee is known as the *pbest*_*i*_ and the best position found so far by all the bees, i.e., the population, is known as the *gbest*. After each bee completes its tasks in each iteration, pheromone laying is done. The bee deposits pheromone using its knowledge of food locations gained so far. To lay pheromone, the *i*^*th*^ bee uses its current location (*X*_*i*_), the best location found by the bee so far (*pbest*_*i*_), and the best location found so far by all the bees (*gbest*). This idea is adopted from Particle Swarm Optimization (PSO) metaheuristic [171], where the local and global best locations are used to update the velocity of the current particle. We have also used the current position in pheromone laying to ensure enough exploration though in MMAS [61] only the current best solution is used to update the pheromone. Only the components which are selected in the corresponding solutions get reinforced. Hence, the pheromone deposition by the *i*^*th*^ bee utilizes Eq. 4 below:

4 $${} \begin{aligned} p_{d}(t+1)&=p_{d} (t)\times w + (r_{0}\times c_{0}\times f_{i} \times x_{X_{i}}^{d})\\ &\quad+(r_{1}\times c_{1}\times pf_{i} \times x_{pbest_{i}}^{d})\\ &\quad+(r_{2}\times c_{2}\times gf \times x_{gbest}^{d}) \end{aligned}  $$

Here, *d*=1,2,⋯,*n* (*n* is the number of genes),*w* is the inertia weight, *f*_*i*_ is the fitness of *X*_*i*_, *pf*_*i*_ is the fitness of *pbest*_*i*_,*gf* is the fitness of *gbest*,$x_{X_{i}}^{d}$ is selection of *d*^*th*^ gene in *X*_*i*_,$x_{pbest_{i}}^{d}$ is selection of *d*^*th*^ gene in *pbest*_*i*_,$x_{gbest}^{d}$ is selection of *d*^*th*^ gene in *gbest*, *c*_0_, *c*_1_, and *c*_2_ determines the contribution of *f*_*i*_, *pf*_*i*_, and *gf* respectively, and *r*_0_, *r*_1_, *r*_2_ are random values in the range of [ 0,1], which are sampled from a uniform distribution.

Here we have, *c*_0_+*c*_1_+*c*_2_=1 and *c*_1_=*c*_2_. So the individual best and the global best influence the pheromone deposition equally. The value of *c*_0_ is set from experimental results presented in the Additional file 1.

The inertia weight is considered to ensure that the contribution of global best and individual best is weighed more in later iterations when they contain meaningful values. To update the value of inertia weight *w*, two different approaches have been considered. One approach updates the weight so that an initial large value is decreased nonlinearly to a small value [37].

5 $$  w(t+1)=\frac{(w(t)-0.4)\times (MAX\_ITER-iter)}{MAX\_ITER+0.4}  $$

Here, *MAX*_*ITER* is the maximum number of iteration and *iter* is the current iteration.

Another approach is to update the value randomly [172].

6 $$ w=\frac {(1+ r_{5})}{2}  $$

Here, *r*_5_ is a random value in the range of [ 0,1], which is sampled from a uniform distribution. Performance evaluation of each of these two approach is presented in the Additional file 1. Pheromone evaporation At the end of each iteration, pheromones are evaporated to some extent. The equation for pheromone evaporation is given by Eq. 7:

7 $$ p_{i}(t+1)=p_{i}(t) \times \rho  $$

Here, (1−*ρ*) is the pheromone evaporation coefficient and *p*_*i*_ is the pheromone corresponding to the *i*^*th*^ gene and *n* is the total number of genes. *p*_*i*_(*t*) represents pheromone value of the *i*^*th*^ gene after (*t*−1)^*th*^ iteration is completed.

Finally, note that, the value of *tmax* is updated whenever a new *gbest* is found. The rationale for such a change is as follows. Over time, as the fitness of *gbest* increases it also contributes more in the pheromone deposition, which may lead the pheromone values for some of the frequent genes to reach *tmax*. At that point, the algorithm will fail to store further knowledge about those particular genes. So we need to update the value of *tmax* after a new *gbest* is found. This is done using Eq. 8 below.

8 $$ tmax(g+1)= tmax(g) \times (1+\rho \times gf)  $$

Here, *tmax*(*g*) represents the value of *tmax* when the *g*^*th*^ global best is found by the algorithm.

**Communication operator** We have incorporated a new operator simulating the communication between the ants in a trail. Even though researchers are unable to establish whether such a communication indeed involves information transfer or not, it is known that foraging decisions of outgoing workers, and their probability to find a recently discovered food source, are influenced by the interactions [62–67]. In fact, there is a large body of evidence emphasizing the role of ant encounters for the regulation of foraging activity particularly for harvester ants [62, 68–71]. Even the mere instance of an encounter may provide information, such as the magnitude of the colony’s foraging activity, and therefore may influence the probability of food collection in ants [72–74].

At each step bees gain knowledge about different components and store their findings by depositing pheromone. After a bee gains new knowledge about the solution components, it share its findings with the successor. So an employed bee gets insight of which components are currently exhibiting excellent performance. Thus a bee obtains idea about food sources from its predecessor. A gene is selected in the current bee if it is selected in its predecessor and pheromone level is greater than a threshold level.

With probability *r*_4_ the following *communication operator* (Eqs. 9 and 10) is applied to each employed bee. The value of *r*_4_ is experimentally tuned and the results are presented in the Additional file 1.

9 $$ x_{X_{i}}^{d}=x_{X_{i-1}}^{d} \times z_{p_{d}}  $$

Where, for *i*^*th*^ bee *i*>1, *d*=1,2,⋯,*n* (*n* is the number of genes), and

10 $$ z_{p_{d}}=\left\{ \begin{array}{ll} 1, & \text{if}~ p_{d} > \frac{tmax}{2} \\ 0, & \text{otherwise} \end{array} \right.  $$

The procedure *Communicate*(*i*) to apply the communication operator on *i*^*th*^ bee is presented in Algorithm 2.



**Initialization** Pheromone for all the genes are initialized to *tmax*. For all the bees food positions are selected randomly. To initialize the *i*^*th*^ bee, the function *initRandom*(*S*_*i*_), given in Algorithm 3, is used. Here we have used a modified sigmoid function that was introduced in [37] to increase the probability of the bits in a food position to be zero. The function is given in Eq. 11 below. It allows the components with high pheromone values to get selected.

11 $$ sigmoid(x)= \frac{1}{1+e^{-x}}  $$

Here, *x*≥0 and *sigmoid*(*x*)∈[0,1]



#### Employed bee phase

To determine a new food position the neighborhood operator is applied to the current food position. Then local search is applied with the probability *probLS* to the new food position to obtain a better position by exploitation. As local search procedures, Hill Climbing (HC), Simulated Annealing (SA), and Steepest Ascent Hill Climbing with Replacement (SAHCR) are considered. Then greedy selection is applied between the newly found neighbor and the current food position. The performance and comparison among different local search methods are discussed in the Additional file 1. In each iteration the value of *gbest*, and *pbest*_*i*_ are updated using the Algorithm 4.



#### Onlooker bee phase

At first a food source is selected according to the goodness of the source using a selection procedure. As the selection procedure, Tournament Selection (TS), Fitness-Proportionate Selection (FPS), and Stochastic Universal Sampling (SUS) have been applied individually and the results are discussed in the Additional file 1. To determine a new food position the neighborhood operator is applied to the food position of the selected bee. Then local search is applied with the probability *probLS* to exploit the food position. As local search methods Hill Climbing, Simulated Annealing, and Steepest Ascent Hill Climbing with Replacement are compared. Then greedy selection is applied between the newly found neighbor and the current food position. In each iteration the value of *gbest*, and *pbest*_*i*_ are updated using the Algorithm 4.

**Selection procedure** In the onlooker bee phase, an employed bee is selected using a selection procedure for further exploitation. As has been mentioned above, tournament selection, fitness-proportionate selection, and stochastic universal sampling have been applied individually as the selection procedure. Tournament selection In this method the fittest individual is selected among the *t* individuals picked from the population randomly with replacement [173], where *t*≥1. Value of *t* is set to 7 in our algorithm. This selection procedure is simple to implement and easy to understand. The selection pressure of the method directly varies with the tournament size. With the increase of the number of competitors, the selection pressure increases. So selection pressure can easily be adjusted by changing the tournament size. If the tournament size is larger, weak individuals have a smaller chance to be selected. The pseudocode is given in Algorithm 5.

 Fitness-proportionate selection In this approach, individuals are selected in proportion to their fitness [173]. Thus, if an individual has a higher fitness, its probability of getting selected is higher. In fitness-proportionate selection which is also known as roulette wheel selection, even the fittest individual may never be selected. In basic ABC, roulette wheel or fitness-proportionate selection scheme is incorporated. The analogy to a roulette wheel can be envisaged by imagining a roulette wheel in which each candidate solution represents a pocket on the wheel; the size of the pockets are proportionate to the probability of selection of the solution. Selecting *N* individuals from the population is equivalent to playing *N* games on the roulette wheel, as each candidate is drawn independently. The pseudocode is given in Algorithm 6.

 Stochastic universal sampling One variant of fitness-proportionate selection is called stochastic universal sampling, which is proposed by James Baker [174]. Where FPS chooses several solutions from the population by repeated random sampling, SUS uses a single random value to sample all of the solutions by choosing them at evenly spaced intervals. This gives weaker members of the population (according to their fitness) a chance to be chosen and thus reduces the unfair nature of fitness-proportional selection methods. In SUS, selection is done in a fitness-proportionate way but biased so that fit individuals always get picked at least once. This is known as a *low variance resampling* algorithm. SUS is used in genetic algorithms for selecting potentially useful solutions for recombination. The method has become now popular in other venues along with evolutionary computation [173]. The pseudocode is given in Algorithm 7.



Other methods like roulette wheel can have bad performance when a member of the population has a really large fitness in comparison with other members. SUS starts from a small random number, and chooses the next candidates from the rest of population remaining, not allowing the fittest members to saturate the candidate space.

#### Scout bee

If the fitness of a bee remains the same for a predefined number (*limit*) of iterations, then it abandons its food position and becomes a scout bee. In basic ABC, it is assumed that only one source can be exhausted in each cycle, and only one employed bee can become a scout. In our modified approach we have removed this restriction. The scout bees are assigned to new food positions randomly. While determining components to form a new food position the solution component with higher pheromone values have higher probability of being selected. The value of *limit* is experimentally tuned and discussed in the Additional file 1. The variable *trial*_*i*_ contains the number of times the fitness remains unchanged consecutively for the *i*^*th*^ bee. The procedure *initRandom*(*S*_*i*_) to assign new food positions for scout bees is given in Algorithm 3. In each iteration the value of *gbest*, and *pbest*_*i*_ are updated using the Algorithm 4.

#### Local search

To explore nearby food sources the basic ABC algorithm applies a neighboring operator to the current food source. But in our algorithm we have applied local search to produce a new food position form the current one. In the employed bee and onlooker bee stages, local search is applied with the probability *probLS* to increase the exploitation ability [95]. The value of *probLS* is scientifically tuned in the Additional file 1. As has already been mentioned above, as the local search procedures, Hill Climbing (HC), Simulated Annealing (SA), and Steepest Ascent Hill Climbing with Replacement (SAHCR) have been employed as the local search procedure. Depending upon the choice *HillClimbing*(*S*) or *SimulatedAnnealing*(*S*) or *SteepestAscentHillClimbingWithReplacement*(*S*) is called form the method *LocalSearch*(*S*). The performance assessment between different local searches and the parameter tuning of the local search methods are discussed in the Additional file 1. Hill climbing Hill climbing is an optimization technique which belongs to the family of local search methods. The algorithm, starting from an arbitrary solution, iteratively tests new candidate solutions in the region of the current solution, and adopt the new ones if they are better. This enables to climb up the hill until local optima is reached. The method does not require to know the strength or direction of the gradient. Hill climbing is good for finding a local optima but it is not necessarily guaranteed to find the global optima. To find a new candidate solution we have applied random tweak to the current solution. The pseudocode is given in Algorithm 8.

 Simulated annealing Annealing is a process in metallurgy where molten metals are slowly cooled to make them reach a state of low energy where they are very strong. Simulated annealing is an analogous optimization method for locating a good approximation to the global optima. It is typically described in terms of thermodynamics. Simulated annealing is a process where the temperature is reduced slowly, starting from mostly exploring by random walk at high temperature eventually the algorithm does only plain hill climbing as it approaches zero temperature. The random movement corresponds to high temperature. Simulated annealing injects randomness to jump out of the local optima. At each iteration the algorithm selects the new candidate solution probabilistically. So the algorithm may sometimes go down hills. The pseudocode is given in Algorithm 9.

 Steepest ascent hill climbing with replacement This method samples all around the original candidate solution by tweaking *n* times. Best outcome of the tweaks is considered as the new candidate solution. The current candidate solution is replaced by the new one rather than selecting the best one between the new candidate solution and the current solution. The best found solution is saved in a separate variable. The pseudocode is given in Algorithm 10.



**Neighborhood operator** In the solution we need only the informative genes to be selected. So we discard the uninformative ones from the solution. By this way we will get a small set of informative genes. To find a nearby food position we first find the genes which are selected in the current position. A number of selected genes (at least one) are dropped from the current solution. We get rid of the genes which tend to appear less potential. If the current solution has zero selected genes then we rather select a possibly informative gene. The parameter *nd* determines the percentage of selected genes to be removed. The value of *nd* is experimentally tuned in the Additional file 1.

Let *X*_*e*_={0,1,1,0,1,0,0,1,1,0,1,1,1,0,0,1,0,1,0,0} is a candidate solution with gene size, *n*=20 and the number of selected gene is 10 (ten). So if *nd*=0.3 we will randomly pick 3 (three) genes which are currently selected in the current candidate solution (*X*_*e*_) and change them to 0. Let the indices 2 (two), 8 (eight), and 15 (fifteen) are randomly selected. So $x_{X_{e}}^{2}$, $x_{X_{e}}^{8}$, and $x_{X_{e}}^{15}$ will become zero. Nearby food position $({X^{n}_{e}})$ of the current candidate solution (*X*_*e*_), found after applying the neighborhood operator will be ${X^{n}_{e}}=\{0, 1, \mathbf {0}, 0, 1, 0, 0, 1, \mathbf {0}, 0, 1, 1, 1, 0, 0, \mathbf {0}, 0, 1, 0, 0\}$ (changes are shown in **boldface** font). Please note that we adopt zero-based indexing.

**Tweak operator** The tweak operation is done by the method *Tweak*(*S*). Here, one of the genes is picked randomly and selection of that gene is flipped. So if the gene is selected, after tweak it will be dropped and vice versa. For example let *X*_*e*_={0,1,1,0,1,0,0,1,1,0,1,1,1,0,0,1,0,1,0,0} is a candidate solution with gene size, *n*=20 and the number of selected gene is 10 (ten). Let randomly the index 6 (six) is selected. So the tweaked food position $({X^{t}_{e}})$ of the current candidate solution (*X*_*e*_), found after applying the tweak operator will be ${X^{t}_{e}}=\{0, 1, 1, 0, 1, 0, \mathbf {1}, 1, 1, 0, 1, 1, 1, 0, 0, 1, 0, 1, 0, 0\}$ (change is shown in **boldface** font). Please note that we adopt zero-based indexing.

**Fitness** Our fitness function has been designed to consider both the classification accuracy and the number of selected genes. The higher the accuracy of an individual the higher is its fitness. On the other hand small number of selected genes yields good solution. So if the percentage of genes that are not selected is higher the fitness will be higher. The value $\frac {n-ns_{i}}{n}$ gives the percentage of genes that are not selected in *S*_*i*_. The tradeoff between weight of accuracy and selected gene size is given by *w*_1_. Higher value of *w*_1_ means accuracy is prioritized more than the selected gene size. So, finally the fitness of the *i*^*th*^ bee (*S*_*i*_) is determined according to Eq. 12.

12 $$ fitness(S_{i})=w_{1} \times accuracy(X_{i})+ (1-w_{1}) \times \frac{n-ns_{i}}{n}  $$

Here, *w*_1_ sets the tradeoff between the importance of accuracy and selected gene size, *X*_*i*_ is the food position corresponding to *S*_*i*_, *accuracy*(*X*_*i*_) is the LOOCV (Leave One Out Cross Validation) classification accuracy using SVM (to be discussed shortly), and *ns*_*i*_ is the number of currently selected genes in *S*_*i*_.

**Accuracy** To assess the fitness of a food position we need the classification accuracy of the gene subset. The predictive accuracy of a gene subset obtained from the modified ABC is calculated by an SVM with LOOCV (Leave One Out Cross Validation). The higher the LOOCV classification accuracy, the better the gene subset. SVM is very robust with sparse and noisy data. SVM has been found suitable for classifying high dimensional and small-sample sized data [142, 175]. Also SVM is reported to perform well for gene selection for cancer classification [20, 176].

The noteworthy implementations of SVM include SVM ^*light*^ [177], LIBSVM [178], mySVM [179], and BSVM [180, 181]. We have included LIBSVM as the implementation of SVM. For a multi-class SVM, we have utilized the OVO (“one versus one") approach, which is adapted in the LIBSVM [178]. The replacement of dot product by a nonlinear kernel function [182] yields a nonlinear mapping into a higher dimensional feature space [183]. A kernel can be viewed as a similarity function. It takes two inputs and outputs how similar they are. There are four basic kernels for SVM: linear, polynomial, radial basic function (RBF), and sigmoid [184]. The effectiveness of SVM depends on the selection of kernel, the kernel’s parameters, and the soft margin parameter *C*. Uninformed choices may result in extreme reduction of performance [142]. Tuning SVM is more of an art than an exact science. Selection of a specific kernel and relevant parameters can be achieved empirically. For the SVM, the penalty factor *C* and *Gamma* are set to 2000, 0.0001, respectively as adopted in Li et al. [41]. Use of linear and RBF kernel and their parameter tuning is discussed in the Additional file 1.

As classifier for both binary class and multi class gene selection methods, use of SVM is present in [23, 37, 41, 42, 45, 54, 153, 157, 164, 165, 185–200].

Cross-validation is believed to be a good method for selecting a subset of features [201]. LOOCV is in one extremity of *k*-fold cross validation, where *k* is chosen as the total number of examples. For a dataset with *N* examples, *N* numbers of experiments are performed. For each experiment the classifier learns on *N*−1 examples and is tested on the remaining one example. In the LOOCV method, a single observation from the original sample is selected as the validation data, and the remaining observations serve as training data. This process is repeated so that each observation in the sample is used once as the validation data. So every example is left out once and a prediction is made for that example. The average error is computed by finding number of misclassification and used to evaluate the model. The beauty of the LOOCV is that despite of the number of generations it will generate the same result each time, thus repetition is not needed.

**Pseudocode for the modified ABC algorithm** Finally, the pseudocode of our modified ABC algorithm used in this article is given in Algorithm 11 and the flowchart of the proposed gene selection method using Algorithm 11 is given in Fig. 1.



## Results and discussion

The algorithm is iterated for *MAX*_*ITER* times to obtain an optimal gene subset. Then the gene subset is classified using SVM with LOOCV to find the accuracy of the subset which gives the performance outcome of a single run. Now to find the performance of our approach and to tune the parameters the algorithm is run multiple times (at least 15 times). Finally the average of accuracy along with the number of selected genes from all the runs for a single parameter combination presents the performance of that parameter combination. In this section the performance of our method will be presented using ten publicly available datasets. Different parameters are tuned to enhance the performance of the algorithm using one of the datasets. Parameter tuning and the contribution of different parameters are discussed in the Additional file 1. Comparison with previous methods that used the same datasets is discussed in this section. We have also presented comparison between different known heuristics methods in this section. Four different parameter settings according to different criteria have been proposed in this paper. Performance comparison for all the parameter settings is given in this section. In all cases the optimal results (maximum accuracy and minimum selected gene size) are highlighted using **boldface** font.

### Datasets

Brief attribute summary of the datasets are presented in Table 1. The datasets contains both binary and multi class high dimensional data. The online supplement to the datasets [192] used in this paper is available at http://www.gems-system.org. The datasets are distributed as Matlab data files (.mat). Each file contains a matrix, the columns consist of diagnosis (1st column) and genes, and the rows are the samples.

**Table 1:** Attributes of the datasets used for experimental evaluation

Name of the dataset	Sample size	Number of genes	Number of classes	Reference
9_*Tumors*	60	5,726	9	[209]
11_*Tumors*	174	12,533	11	[196]
*Brain*_*Tumor*1	90	5,920	5	[210]
*Brain*_*T*_ *umor*2	50	10,367	4	[211]
*DLBCL*	77	5,469	2	[212]
*Leukemia*1	72	5,327	3	[5]
*Leukemia*2	72	11,225	3	[213]
*Lung*_*Cancer*	203	12,600	5	[214]
*Prostate*_*Tumor*	102	10,509	2	[215]
*SRBCT*	83	2,308	4	[216]

### Optimized parameter values

While selecting the optimized parameter setting (Table 5) we have considered other factors besides the obtained performance.

After analyzing the results (Table S3 in Additional file 1), we have decided to use 0.5 as the value of *r*_4_ in our final experiments. Probability value of 0.7 for local search has been used to ensure that too much exploitation is not done despite that the value of 1.0 gives the highest accuracy (Table S5 in Additional file 1). The value of *nd* is set to 0.035 as it demonstrates a good enough accuracy with tolerable gene set size among all the values considered for the parameter (Table S6 in Additional file 1). Population size is kept at 25 which shows an acceptable level of accuracy (Table S9 in Additional file 1). We have selected SAHCR as the local search method at the onlooker bee stage and SA at the employed bee stage to ensure both exploration and exploitation. The value 12 is set as iteration count of SAHCR as it shows acceptable accuracy (Table S19 in Additional file 1). The value is kept small because increased iteration count increases the algorithm running time. The value 9 is considered as the final value for number of tweaks in SAHCR (Table S20 in Additional file 1). The value 0.065 is selected as the percentage of genes to be preselected in the preprocessing stage despite that the value 0.03 gives the best accuracy (Table S21 in Additional file 1). This is done because choosing 0.03 might possess the risk of discarding informative genes in the prefiltering step for other datasets. The value 0.6 is set for *c*_0_ as it shows good results (Table S23 in Additional file 1). The obtained accuracy is highest for the *limit* value 100 (Table S25 in Additional file 1). But high value of *limit* may result in less exploration. Thus, we recommend *limit* = 35 for this parameter setting after considering the experimental outcomes. Among TS and FPS, TS is considered as the selection method.

The optimized parameter values are listed in Table 2. From the obtained results (Table 7) we can conclude that the algorithm performs consistently for all datasets based on the standard deviation for accuracy (maximum 0.01) and number of selected gene (maximum 5.64) for optimized parameter settings. Our algorithm in fact has achieved satisfactory accuracy even for the default parameter settings albeit with a high standard deviation for the number of selected genes for most of the cases. The main reason for high standard deviation in the selected gene size for the default parameter setting can be attributed to the high default value of *c*_0_ and low default value of *limit*.

**Table 2:** Optimized parameter values after tuning

Parameter	Optimized value
*probLS*	0.7
*ρ*	0.8
*w*	1.4
*w*_1_	0.85
*th*_*n*_	0.065
*sahc*_*iter*	12
*sahc*_*tweak*	9
*sa*_*iter*	14
*t*	5
*schedule*	0.5
*tmax*	5
*tmin*	0
*c*_0_	0.6
*MAX*_*ITER*	20
*limit*	35
*nd*	0.035
*PS*	25
*r*_4_	0.5
*ls*_*e*_	SA
*ls*_*o*_	SAHCR
Selection method	Tournament selection
*kernel*	Linear
*wt*	Equation 5
*uph*	True
*prefilter*	Kruskal-Wallis

**Fig. 2:**
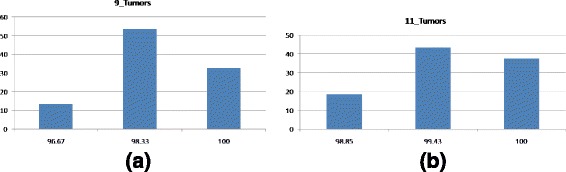
Distribution of classification accuracy using first (optimized) parameter setting for the dataset **a** 9_*Tumors*; **b** 11_*Tumors*

**Fig. 3:**
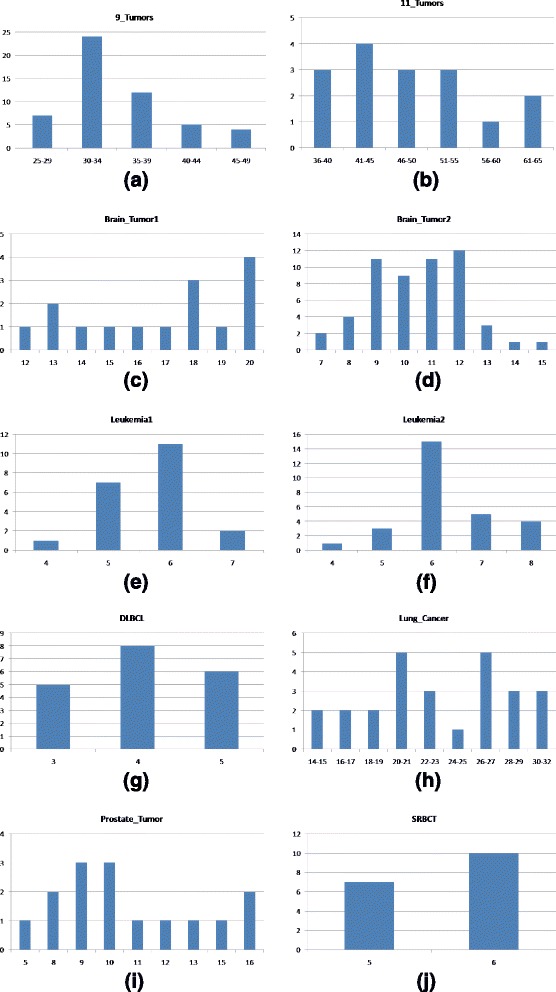
Distribution of number of times selected gene size fall in a specific range using the first (optimized) parameter setting **a** 9_*Tumors*; **b** 11_*Tumors*; **c** *Brain*_*Tumor*1; **d** *Brain*_*Tumor*2**e** *Leukemia*1; **f** *Leukemia*2; **gDLBCL**; **h** *Lung*_*Cancer*; **i** *Prostate*_*Tumor*; **jSRBCT**

The Fig. 2 shows the distribution of obtained accuracy in optimized parameter settings for the dataset 9_*Tumors* and 11_*Tumors*. For all other datasets our method obtained 100 *%* accuracy in all the runs. The horizontal axis represents the accuracy and the vertical axis represents the percentage of time corresponding accuracy is obtained among all the runs. Similarly the Fig. 3 represents the distribution of selected gene size in optimized parameter settings for all the datasets. The horizontal axis represents the selected gene size and the vertical axis represents the percentage of time corresponding gene size is obtained among all the runs.

### Comparison with different metaheuristics

To compare our method with different metaheuristics we have considered ABC, GA, and ACO. In Additional file 1 performance of GA and ACO respectively for different parameter combination is discussed. Finally, the tuned parameter values are considered to run the experiments for comparing with our proposed method. For ABC, optimized parameter values found for mABC are considered (Table S31 in Additional file 1). Table 3 shows comparison between our work and the evolutionary algorithms in consideration. For this comparison we have considered the 9_*Tumors* dataset. The results are presented in Table 3. From the results we can see that ABC performs significantly better than GA and ACO. Our experimental results support the study done by Karaboga et al. [86]. Finally, from Table 3 we can see that our proposed mABC performs better than other metaheuristics in consideration according to both the constraints.

**Table 3:** Comparative experimental results of the best subsets produced by mABC and other evolutionary methods for the dataset 9_*Tumors*

Evaluation	GA					ACO					ABC					mABC [This work]			
Criteria	Best	Avg.	S.D.	Worst		Best	Avg.	S.D.	Worst		Best	Avg.	S.D.	Worst		Best	Avg.	S.D.	Worst
Accuracy	85	82.56	**0.01**	81.67		85	83.09	**0.01**	81.67		91.67	87.67	0.02	85		**100**	**98.65**	**0.01**	**96.67**
# Genes	275	276.8	12.07	301		266	271.59	11.03	279		57	75.67	14.18	97		**30**	**34.73**	**5.64**	**35**

Best results (maximum accuracy and minimum selected gene size) are highlighted using **boldface** font

### Comparison with existing methods

Table 4 shows comparison between our work and other works in literature including EPSO for the datasets 9_*Tumors*, 11_*Tumors*, *Brain*_*Tumor*1, *Brain*_*Tumor*2, *DLBCL*, *Leukemia*1, *Leukemia*2, *Lung*_*Cancer*, *Prostate*_*Tumor*, and *SRBCT*. The optimized parameter setting listed in Table 2 are used to run the algorithm for at least 15 times. Obtained accuracy (both average and best) by our approach for all the datasets are better or at least equal to the accuracy achieved by EPSO.

**Table 4:** Comparative experimental results of the best subsets produced by mABC and other methods for different datasets

Dataset name	Evaluation	EPSO				IBPSO		TS-BPSO		BPSO-CGA		Random forest			mABC		
	Criteria	[37]				[43]		[42]		[44]		[55]			[This work]		
		Best	Avg.	S.D.		Best		Best		Best		Avg.	S.D.		Best	Avg.	S.D.
9_*Tumors*	Accuracy	76.67	75.00	1.11		78.33		81.63		-		88.0	0.037		**100**	**98.65**	**0.01**
	# Genes	251	247.10	9.65		1280		2941		-		130	-		**30**	**34.73**	**5.64**
11_*Tumors*	Accuracy	96.55	95.40	0.61		93.10		97.35		-		-	-		**100**	**99.50**	**0.00**
	# Genes	243	237.70	9.66		2948		3206		-		-	-		**42**	**47.27**	**7.79**
*Brain*_*Tumor*1	Accuracy	93.33	92.11	0.82		94.44		95.89		91.4		93.6	0.007		**100**	**100**	**0.0**
	# Genes	**8**	**7.5**	**2.51**		754		2913		456		86	-		12	16.87	2.85
*Brain*_*Tumor*2	Accuracy	94	92.4	1.27		94.00		92.65		-		-	-		**100**	**100**	**0.0**
	# Genes	4	6.0	1.83		1197		5086		-		-	-		**7**	**10.52**	**1.72**
*DLBCL*	Accuracy	**100**	**100**	**0**		**100**		**100**		-		94.6	0.021		**100**	**100**	**0.0**
	# Genes	**3**	4.7	0.82		1042		2671		-		21	-		3	4.05	0.78
*Leukemia*1	Accuracy	**100**	**100**	**0**		**100**		**100**		**100**		98.1	0.006		**100**	**100**	**0.0**
	# Genes	**2**	**3.20**	**0.63**		1034		2577		300		21	-		4	5.67	0.73
*Leukemia*2	Accuracy	**100**	**100**	**0**		**100**		**100**		-		-	-		**100**	**100**	**0.0**
	# Genes	**4**	6.8	2.2		1292		5609		-		-	-		**4**	**6.29**	**0.98**
*Lung*_*Cancer*	Accuracy	96.06	95.67	0.31		96.55		99.52		-		-	-		**100**	**100**	**0.0**
	# Genes	**7**	**8.3**	**2.11**		1897		6958		-		-	-		14	23.31	5.14
*Prostate*_*Tumor*	Accuracy	99.02	97.84	0.62		92.16		95.45		93.7		-	-		**100**	**100**	**0.0**
	# Genes	**5**	**6.6**	**2.17**		1294		5320		795		-	-		**5**	10.73	3.15
*SRBCT*	Accuracy	**100**	99.64	0.58		**100**		**100**		**100**		99.7	0.003		**100**	**100**	**0.0**
	# Genes	7	14.90	13.03		431		1084		880		42	-		5	5.59	0.51

Best results (maximum accuracy and minimum selected gene size) are highlighted using **boldface** font

For 9_*Tumors*, with the optimized parameter values, our algorithm has achieved 100 *%* accuracy in 32.7 *%* runs (Fig. 2a). Average accuracy obtained by this work for this dataset is significantly better than EPSO. Also number of selected genes to achieve the accuracy is remarkably lower than EPSO. Our method selected at least 73.28 *%* less genes than other methods. It may appear that the reason for exceptionally better performance for 9_*Tumors* dataset is that the parameter values are optimized specially for this dataset. But even the worst performances for both default and optimized parameter values (Table 7) by this work are better than that of EPSO. For the dataset 11_*Tumors*, in 23.8 *%* runs our method obtained the highest (100 *%*) accuracy (Fig. 2b). The average accuracy obtained by our approach is better than other methods. The average no. of selected genes size is significantly better than previous methods. Our approach obtained at least 40.36 *%* less gene than previous methods. For *Brain*_*Tumor*1, and *Brain*_*Tumor*2 the obtained accuracy is better than EPSO and other methods with 100 *%* accuracy in all the runs. But the number of selected gene on average is little higher than EPSO. For *DLBCL* both our works and EPSO have achieved 100 *%* accuracy with 0 standard deviation. But on average number of selected gene is smaller in our algorithm though the best result by both the approaches are the same. For *Leukemia*1, and *Leukemia*2 our method has achieved highest (100 *%*) accuracy like EPSO. But our obtained marginally larger amount of genes than EPSO in the best obtained result for *Leukemia*1 (2 more). And for *Leukemia*2 our proposed method selected same number of genes as EPSO in the best obtained result. Also for *Leukemia*2 dataset, average number of genes selected is smaller in this work. For *Lung*_*Cancer* dataset, our algorithm achieved the highest (100 *%*) accuracy which is better than other methods. But the selected gene size for both the best and the average result is little higher than EPSO. For the dataset *Prostate*_*Tumor*, this work has exhibited better performance according to accuracy. Our method has obtained highest (100 *%*) accuracy with zero standard deviation which is the best accuracy obtained so far by any methods. But average number of selected genes is little higher in our method though best selected gene size is same as EPSO. For *SRBCT* our method has shown better performance in all cases (according to both accuracy and the number of selected genes for both optimized and default parameter values). For this dataset best result achieved by our method selected only 5 genes (better than EPSO) while obtained 100 *%* accuracy (same as EPSO). Even the worst results obtained by our algorithm in optimized parameter setting (Table 2) is better than the best result achieved by EPSO. Also our method exhibits more consistent performance according the lower standard deviation than EPSO for both accuracy (maximum 0.01) and selected gene size (maximum 5.64) for most of the datasets. So, in summary, for all the datasets gained accuracy and standard deviation of accuracy by our method is better or equal to the accuracy obtained by EPSO. However, for some cases the number of selected genes is a little higher. To obtain the stated results we have used only 20 iterations while EPSO used 500 iterations [37].

### Further tuning of parameters

Tuning with full factorial combination would have allowed us to find the best parameter settings. But it will require enormous computational time. To demonstrate this hypothesis we have done the experiments using another two parameter settings (Table 5) formed from two different viewpoints. While selecting the optimized parameter setting (Table 2) we have considered many other factors besides the performance. So, we have configured two other parameter settings where the performance is considered as the major criterion of value selection along with running time. The last parameter settings (given in Table 5) is created after further tuning is done. Comparison between all the parameter settings including the default one is given in Table 7. From the obtained results (Table 7) it is clear that further tuning can improve results for all the datasets.

**Table 5:** All the proposed parameter values after tuning

Parameter	First	Second	Third
*probLS*	0.7	0.4	0.7
*ρ*	0.8	0.8	0.8
*w*	1.4	1.4	1.4
*w*_1_	0.85	0.85	0.85
*th*_*n*_	0.065	0.03	0.03
*sahc*_*iter*	12	16	16
*sahc*_*tweak*	9	15	15
*hc*_*iter*	N/A	10	N/A
*sa*_*iter*	10	N/A	N/A
*t*	5	N/A	N/A
*schedule*	0.5	N/A	N/A
*tmax*	5	5	5
*tmin*	0	0	0
*c*_0_	0.6	0.5	0.5
*MAX*_*ITER*	20	20	20
*limit*	35	100	100
*nd*	0.035	0.02	0.035
*PS*	25	40	40
*r*_4_	0.5	0.7	0.7
*ls*_*e*_	SA	HC	SAHCR
*ls*_*o*_	SAHCR	SAHCR	SAHCR
Selection method	Tournament selection	Tournament selection	Stochastic universal sampling
*kernel*	Linear	Linear	Linear
*wt*	Equation 5	Equation 5	Equation 5
*uph*	True	True	True
*prefilter*	Kruskal-Wallis	Kruskal-Wallis	Kruskal-Wallis

#### Second parameter setting

To propose the second parameter setting we have considered the performance as the major criterion along with the running time for selecting parameter values. High probability of local search increases performance (Table S5 in Additional file 1). But higher probability will also result in increased running time and little exploration. So while preparing this parameter combination we decided to keep the probability low allowing enough exploration, but other values are selected considering the performance outcome mainly. The probability value of 0.4 is selected for local search to prevent too much exploitation and decrease running time. The value 0.03 is selected as the percentage of genes to be preselected in the preprocessing because it gives the best accuracy. The value 0.6 is set for *c*_0_ as it shows the best results. The obtained accuracy is highest for the *limit* value 100 which is selected for this parameter. The value of *nd* is set to 0.02 as it demonstrates a good enough accuracy. Population size is increased to 40 which gives the best accuracy and small number of selected genes. The value of *r*_4_ is increased to 0.7, as increased application of communication operator improves the results. HC is selected as local search method in employed bee stage and SAHCR is selected as local search method in onlooker bee stage because this combination needs comparatively less running time but gives considerably good results. Also the iteration and tweak count for the local search method SAHCR is increased. The main idea behind proposing this parameter setting is to improve performance than the previous parameter setting with decreased running time. So we have selected low probability of local search but for other parameters mainly we have selected values which give best performance.

#### Third parameter setting

The third parameter settings is proposed after further tuning is done for one of the parameters named “Selection method". To find if further performance upgrade is possible by considering new values we have considered another selection method named Stochastic Universal Sampling (SUS). The results are given in Table 6. From the results we can see that the newly considered method SUS performs better than the others.

**Table 6:** Performance outcome for different values of parameter selection method

**Values**	**Accuracy**		**No. of selected gene**	
	Avg.	S.D.	Avg.	S.D.
Fitness proportionate selection	84.2	**0.03**	41.43	47.47
Tournament selection	84.74	**0.03**	53.33	53.65
Stochastic universal sampling	**85.42**	**0.03**	**35.19**	**5.49**

Best results (maximum accuracy and minimum selected gene size) are highlighted using **boldface** font

So, finally, we have proposed another parameter settings which considers the performance as the main criterion to select the parameter values. So, we have kept the probability of the local search high (0.7). This setup takes the highest time to run. Because the probability of local search is kept high and SAHCR is used as local search method for both the stages. SAHCR as local search for both the stages performs the best (Table S14 in Additional file 1). SUS is used as the selection method in onlooker bee stage. Other parameter values are same as optimized (first) parameter setting. The parameter settings is given in Table 5.

#### Comparison between different parameter settings

Comparison between all the parameter settings including the default one is given in Table 7. For all the parameter settings the best, average, standard deviation (S.D.), and the worst results are reported. First we will present the comparison between results achieved by the default and the first parameter settings. Next we will compare the results obtained by second and third parameter settings with the first parameter settings.

In all cases the first parameter setting exhibits better results according to both accuracy and the number of selected genes than the default parameter setting. For the first parameter setting we can conclude that the algorithm performs consistently for all datasets based on the standard deviation for accuracy (maximum 0.01) and number of selected gene (maximum 5.64). Our algorithm in fact has achieved satisfactory accuracy even for the default parameter setting albeit with a high standard deviation for the number of selected genes for most of the cases. The main reason for high standard deviation in the selected gene size for the default parameter setting can be attributed to the high default value of *c*_0_ and low default value of *limit*.

The best, average, worst, and standard deviation obtained using the first, second, and third parameter settings for all the datasets are given in Table 7. Now we will present comparison between these parameter settings. For the 9_*Tumors* dataset, best obtained accuracy for all the parameter settings is same (100 % accuracy). But the selected gene size (21) for the best results are same for newly proposed two parameter settings which is better (30 % lower) than the selected gene size (30) obtained by the proposed first parameter settings. For the 11_*Tumors* dataset obtained average accuracy by third parameter setting is better than other parameter settings. Best obtained accuracy by all three parameter setting is 100 %. But selected gene size for best result is better in the second parameter settings. Also the second parameter setting selected lower (at least 9.18 % lower) number of genes on average than others. Second and third proposed parameter settings selected much lower (at least 33.89 % lower) number of genes on average than the first parameter setting for this dataset. For all other datasets obtained accuracy by all three parameter settings in all the runs is 100 %. For the dataset *Brain*_*Tumor*1, the best and the average number of selected genes are better for the third parameter setting. Last two parameter settings obtained at least 33.61 % lower selected genes size than the average number of selected genes by the first parameter setting. The average number of selected genes by the third parameter setting is better (5.54 % lower) than the average number of genes selected by the second parameter setting. And the maximum number of selected genes (13) is same for the last two parameter settings. For the dataset *Brain*_*Tumor*2, minimum (6) and maximum (9) number of selected genes are same for the second and third parameter settings. But selected gene size on average is smaller (1.61 % lower) for the third parameter settings than the average selected gene size by the second parameter settings. For both the second and third parameter settings selected gene size on average is lower (at least 28.99 % lower) than that of first parameter setting. For the dataset *DLBCL*, minimum number of selected gene size (3) is same for all three parameter settings. But average selected gene size obtained by the last two parameter combinations are at least 11.11 % lower than the average selected gene size obtained by the first parameter setting. The maximum number of genes selected (4) by the second and the third parameter settings are same. But on average selected gene size by the third parameter setting is 7.5 % lower than the average number of genes selected by the second parameter setting. For the *Leukemia*1 dataset, best minimum number of selected genes (3) is obtained by the third parameter setting. On average the last two proposed parameter settings selected at least 22.4 % lower number of genes than the first parameter setting. Also the average number of genes selected by the third parameter setting is 12.05 % lower than the average number of selected genes by the second parameter setting. For the dataset *Leukemia*2, minimum number of selected genes (3) by both the second and the third parameter setting is same, which is better than the minimum number of selected genes (4) by the first parameter setting. Also the maximum number of selected genes (5) by the second and the third parameter setting are the same, which is lower than the maximum number of genes selected (8) by the first parameter setting. Average number of selected genes by the second and third parameter settings is at least 35.45 % lower than the average selected gene size by the first parameter setting. But for this datasets the average number of selected gene size is minimum for the second parameter setting, which is 4.68 % lower than the average number of selected gene size by the third parameter setting. For the *Lung*_*Cancer* dataset, minimum number of selected genes (9) by the second and the third parameter settings is 35.71 % smaller than the minimum number of selected genes (14) obtained by the first parameter setting. Also the maximum number of selected genes (14) by the second and the third parameter settings is 56.25 % lower than the maximum number of selected genes (32) by the first parameter setting. Note that the best obtained gene set size (14) by the first parameter setting for this dataset is same as the worst obtained gene set size (14) by the last two proposed parameter combinations. Also the second and third parameter settings obtained average selected gene size at least 46.63 % smaller than the average number of selected genes by the first parameter setting. But the average selected gene size by the second parameter setting is better (8.12 % lower) than the average selected gene size by the third parameter setting. For the *Prostate*_*Tumor* dataset, the minimum number of selected genes (5) is same for all the parameter settings. But the worst obtained gene subset size (8) by the second and the third parameter settings is better (50 % lower) than the worst obtained gene subset size (16) by the first parameter setting. Also the average number of selected genes by the first parameter setting is at least 62.82 % higher than the average number of selected genes by the last two parameter settings. Again the average number of genes selected by the third parameter setting is better (1.37 % lower) than the average number of genes selected by the second parameter setting. For the dataset *SRBCT*, minimum number of genes selected (4) by the last two parameter setting is better than the minimum number of genes selected (5) by the first parameter setting. The average number of genes selected by the second and third parameter settings are at least 23.61 % better than the average number of genes selected by the first parameter setting. Moreover selected gene size by the third parameter setting is same in all the run, thus standard deviation is zero. So average number of selected genes for the third parameter setting is 6.32 % lower than the average number of genes selected by the second parameter setting. For the datasets 9_*Tumors*, 11_*Tumors*, *Leukemia*2, and *Lung*_*Cancer* obtained average number of selected genes is better for the second parameter setting. In all other cases considering both the accuracy and the selected gene size the third parameter setting performed comparatively better.

**Table 7:** Experimental results of mABC using default, optimized (first), second and third parameter settings for different datasets

Dataset name	Evaluation	Default					PS1					PS2					PS3			
	Criteria	Best	Avg.	S. D.	Worst		Best	Avg.	S. D.	Worst		Best	Avg.	S. D.	Worst		Best	Avg.	S. D.	Worst
9_*Tumors*	Accuracy	90.0	84.60	0.02	80.0		**100**	98.65	**0.01**	96.67		**100**	99.11	**0.01**	**98.33**		**100**	**99.63**	**0.01**	**98.33**
	# Genes	41	50.03	52.21	37		30	34.73	5.64	35		**21**	**24**	4	26		**21**	25.5	**3.75**	**24**
11_*Tumors*	Accuracy	94.25	93.08	0.01	91.38		**100**	99.50	**0.0**	98.85		**100**	99.45	**0.0**	98.85		**100**	**99.7**	**0.0**	**99.43**
	# Genes	33	37.15	5.08	23		42	47.27	7.79	47		**30**	**30.75**	4.15	**30**		32	33.86	**3.44**	34
*Brain*_*Tumor*1	Accuracy	100	95.85	0.02	93.33		**100**	**100**	**0.0**	**100**		**100**	**100**	**0.0**	**100**		**100**	**100**	**0.0**	**100**
	# Genes	13	16.53	5.78	16		12	16.87	2.85	20		10	11.2	**1.08**	**13**		**8**	**10.58**	1.17	**13**
*Brain*_*Tumor*2	Accuracy	100	98.09	0.01	96		**100**	**100**	**0.0**	**100**		**100**	**100**	**0.0**	**100**		**100**	**100**	**0.0**	**100**
	# Genes	11	17.6	5.82	16		7	10.52	1.72	15		**6**	7.47	1.19	**9**		**6**	**7**.35	**1.0**	**9**
*DLBCL*	Accuracy	100	100	0	100		**100**	**100**	**0.0**	**100**		**100**	**100**	**0.0**	**100**		**100**	**100**	**0.0**	**100**
	# Genes	4	7.67	1.77	11		**3**	4.05	0.78	5		**13**	3.6	0.51	**4**		**3**	**3.33**	**0.49**	**4**
*Leukemia*1	Accuracy	100	100	0	100		**100**	**100**	**0.0**	**100**		**100**	**100**	**0.0**	**100**		**100**	**100**	**0.0**	**100**
	# Genes	5	8.39	1.94	12		4	5.67	0.73	7		4	4.4	0.51	5		**3**	**3.87**	**0.35**	**4**
*Leukemia*2	Accuracy	100	100	0	100		**100**	**100**	**0.0**	**100**		**100**	**100**	**0.0**	**100**		**100**	**100**	**0.0**	**100**
	# Genes	6	10.64	2.57	15		4	6.29	0.98	8		**3**	**3.87**	0.74	**5**		**3**	4.06	**0.64**	**5**
*Lung*_*Cancer*	Accuracy	99.01	98.17	0.01	97.04		**100**	**100**	**0.0**	**100**		**100**	**100**	**0.0**	**100**		**100**	**100**	**0.0**	**100**
	# Genes	27	24.59	6.94	16		14	23.31	5.14	32		**9**	**11.43**	**1.34**	**14**		**9**	12.44	1.5	**14**
*Prostate*_*Tumor*	Accuracy	100	96.95	0.01	95.10		**100**	**100**	**0.0**	**100**		**100**	**100**	**0.0**	**100**		**100**	**100**	**0.0**	**100**
	# Genes	11	15.33	6.35	19		**5**	10.73	3.15	16		**5**	6.59	0.91	**8**		**5**	**6.5**	**0.82**	**8**
*SRBCT*	Accuracy	100	100	0	100		**100**	**100**	**0.0**	**100**		**100**								
textbf100	**0.0**	**100**		**100**	**100**	**0.0**	**100**													
	# Genes	6	7.47	0.94	9		5	5.59	0.51	6		**4**	4.27	0.46	5		**4**	**4**	**0.0**	**4**

Best results (maximum accuracy and minimum selected gene size) are highlighted using **boldface** font

### Conclusions

Microarray technology allows producing databases of cancerous tissues based on gene expression data [202]. Available training datasets for cancer classification generally have a fairly small sample size compared to the number of genes involved and consists of multiclass categories. The sample size is likely to remain small at least for the near future due to the expense of microarray sample collection [203]. The huge number of genes causes grave computational overhead and poor predictive accuracy in wrapper methods when searching for significant genes. So to select small subsets of relevant genes involved in different types of cancer remains a challenge. So we apply a statistical method in preprocessing step to filter out the noisy genes. Then a search method is utilized for further selection of smaller subset of informative genes. Selection of pertinent genes enable researchers to obtain significant insight into the genetic nature of the disease and the mechanisms responsible for it [183, 204]. Recent research has demonstrated that one of the most important applications of microarrays technology is cancer classification [205, 206]. Biomarker discovery in high-dimensional microarray data helps studying the biology of cancer [207]. When a large number of noisy, redundant genes are filtered the performance of cancer classification is improved [208]. Besides, gene selection can also cut down the cost of medical diagnoses. We believe that the selection of genes by our system provide us some interesting clue towards the importance and contribution of that set of particular genes for the respective cancer disease. To elaborate, our system has identified that for diffuse large B-cell lymphoma (DLBCL) only three (3) genes are informative enough to decide about the cancer. Now, this could turn out to be a string statement with regards to the set of genes identified for a particular cancer and we believe further biological validation is required before making such a string claim. We do plan to work towards validation of these inferences. To this end we believe that our method presented in this paper is a significant contribution and would be useful in medical diagnosis as well as for further research.

## Additional file

Additional file 1 Gene Selection for Cancer Classification With the Help of Bees. The supplementary file is available in the link: https://goo.gl/APTj0n. (PDF 417 kb)
