# Supplementary material for: Gene selection for cancer classification with the help of bees
Source: BMC Med Genomics. 2016 Aug 10;9(Suppl 2):47. doi: 10.1186/s12920-016-0204-7 (PMC4980787; doi:10.1186/s12920-016-0204-7)
Supplement: Additional file 1 — Gene Selection for Cancer Classification With the Help of Bees. The supplementary file is available in the link: https://goo.gl/APTj0n. (PDF 417 kb) [file 12920_2016_204_MOESM1_ESM.pdf]

RESEARCH

Gene selection for cancer classification with the help of bees

Johra Muhammad Moosa[1*], Rameen Shakur[2], Mohammad Kaykobad[1] and Mohammad Sohel Rahman[1]

*Correspondence:
johra.moosa@gmail.com
[1]AℓEDA Group, Department of CSE, BUET, Dhaka-1205 Dhaka, Bangladesh
Full list of author information is available at the end of the article

Parameter Tuning

Different parameters have been experimentally tested for different values. For each parameter value we ran the algorithm for at least 15 times. During these runs, all the parameter values except for the parameter to be tuned are set to default. The default values are given in Table S1. The average and standard deviation of all the runs represent the performance of a specific value. The default parameter values are set from intuition and preliminary runs. The parameter value for which average fitness is maximum has been be picked as optimal parameter value unless stated otherwise. Full factorial combination for parameter values is avoided assuming that the parameters are not interdependent. For tuning the parameters, one of the datasets (9_Tumors) among the ten is selected. The reason to select this dataset is that it is a multiclass dataset and its gene size ($5,726$) is neither too large nor too small compared to the others (please refer to Table S2). Also it has moderate sample size (60). So it is anticipated that the behavior of the parameters for this dataset will more or less be reflected in all other datasets. The analysis of the tuned parameters are discussed in the subsequent sections.

Brief attribute summary of the datasets are presented in Table S2. The datasets contain both binary and multi class high dimensional data. The online supplement to the datasets [1] used in this paper is available at `http://www.gems-system.org`. The datasets are distributed as Matlab data files (.mat). Each file contains a matrix, the columns consist of diagnosis (1st column) and genes, and the rows are the samples.

Probability of Applying the Communication Operator, r_4 To analyze this parameter values from $\{0.0, 0.1, 0.3, 0.5, 0.7, 1.0\}$ have been used. The value 0.0 means running the algorithm without applying the communication operator. On the other hand the value 1.0 means applying the operator in each iteration. The experimental results are presented in Table S3. From the experimental data we can see that use of communication operator increases the accuracy while number of selected genes tends to increase a little. With the increase of probability of utilizing the communication operator there is also an increase in accuracy. So this experiment indicates that the communication operator indeed guides the bees in finding solution with higher accuracy.

Use of Pheromone, uph To understand the contribution of pheromone in our algorithm we need to observe the algorithm performance both with and without using

pheromone. Table S4 presents performance of the algorithm with and without using pheromone. The accuracy remains almost unchanged with the use of pheromone. But from the experimental results we can see that there is an enormous reduction in the number of selected genes. The reason behind this improvement is that the pheromone emphasizes the informative genes found in previous iterations. Much more iterations will be needed to reach the target fitness if we rely mostly on random exploration.

Probability of Local Search, probLS Values from 0 to 1, with step size 0.1 are tested to tune this parameter. The results are listed in Table S5. The relation between change in accuracy with $probLS$ is presented in the Fig. S1. And the relation between change in selected gene size with $probLS$ is exhibited in the Fig. S2. From the experimental values it is observed that with the increase of the probability of employing local search, accuracy of the algorithm also increases. Selected gene size tends to decrease with increasing probability of local search. Note also that more application of the local search increases the running time.

Neighborhood Operator Destruction Size, nd For tuning the parameter nd, values from $\{0.001, 0.005, 0.01, 0.015, 0.02, 0.025, 0.03, 0.035, 0.04, 0.05, 0.06, 0.08, 0.1, 0.15, 0.2\}$ have been considered. The experimental results are presented in Table S6. The accuracy seems to improve with higher values of nd. This is because large value of nd means that large number of genes will be removed from the individual which also increases the probability of removing more noisy genes. But high value of nd results in high number of selected genes, because, too little or too big a jump at a time fails to find a potential neighbor. So, if nd is too large, finding potential candidate after reducing the gene subset will be less frequent. As a result the algorithm fails to achieve a small gene subset.

Pheromone Persistence Factor, ρ The amount of pheromone to be retained from previous iterations is determined by ρ. So, $(1 - \rho)$ is known as the pheromone evaporation coefficient. In this experiment, the parameter ρ takes values from 0 to 1, with step size 0.1 are tested to tune this parameter. The performance for different parameter values for ρ is presented in Table S7. The experimental outcome shows that an increase in the value of ρ results in an increase in both accuracy (upto 0.8) and selected gene set size. In each iteration, $(1 - \rho) \times 100\%$ of the current pheromones are evaporated. So increase in ρ means less evaporation and thus, pheromone containing history of the previous iterations can retain longer. So good solution components will contain high pheromone values for more iterations as previous knowledge is remembered for much longer period of time. As a result during the gene selection, they are more likely to be selected. Hence, bees ability to use their experience while selecting genes also strengthens which results in higher accuracy. Again redundant and noisy genes that get selected also contributes for longer period of time which results in higher selected genes size. The value 0.8 is set as the tuned value for ρ as it shows the highest accuracy. When no pheromone is evaporated (ρ is 1.0), the algorithm selects lowest number of genes. As the information about all the components are stored and thus the most informative genes are detected. But also for this value the obtained accuracy is lowest because of stagnation.

Weight of Accuracy in Fitness, w_1 The tradeoff between accuracy and the number of selected genes is controlled by the parameter w_1. In the fitness equation, w_1 is the weight for the accuracy whereas $(1 - w_1)$ is the weight for the number of selected genes. To tune the parameter w_1, values from $\{0.5, 0.6, 0.65, 0.7, 0.75, 0.8, 0.85, 0.9\}$ are considered. Results of using different parameters are given in Table S8. The Fig. S3 represents a graph showing change in accuracy with respect to change in w_1. The graph shows increasing trend of accuracy. It reaches its peak at the value of 0.85 for w_1. For further values accuracy remains almost same. Increase in w_1 results in increase in accuracy, which is expected because increase in w_1 means accuracy is weighted more. The Fig. S4 represents the graph showing the behavior of selected gene size with respect to change in w_1. Also increase of w_1 means selected gene size weighs less in fitness function. So for higher values of w_1 the number of selected gene is also high. From the graphs (Fig. S3 and S4) it is confirmed that the algorithm perform best in the range of 0.7 to 0.85 considering both the constraints. The value 0.85 is selected as optimized value as it gives the highest accuracy.

Population Size, PS The values in the range of 10 to 50 with step size 5, are considered for tuning the parameter PS. Experimental results using different parameter values are given in Table S9. Notable increase in PS shows negligible increase in accuracy. Also a slight reduction of the selected gene set size is noticed up to a value of 30. For population size 30, the number of selected gene is noticeably small but again the value increases for higher population size. For population size 40 highest accuracy is achieved.

Prefiltering Method We have considered two prefiltering methods: one is parametric (F-test) and the other is non-parametric (Kruskal-Walllis). The performance of application of these two methods in our approach is reported in Table S10. Kruskal-Wallis method shows better performance according to accuracy. This is expected because Kruskal-Wallis is a nonparametric method which is known to be suitable for gene selection [2, 3]. On the other hand, F-test seems to filter out the redundant genes more effectively resulting in relatively smaller selected gene size (at least for the dataset we used for parameter tuning, i.e., 9_Tumors). The performance of the filtering methods depends on whether the dataset contains normal distribution or not. So further study may consider choosing prefiltering method depending upon the result of checking the normality of the dataset. We have used Kruskal-Wallis method in our final experiments.

Selection Method at the Onlooker Bee Stage As the selection procedure at the onlooker bee stage, Tournament Selection and Fitness-Proportionate Selection have been reviewed. The experimental results are presented in Table S11. Fitness-proportionate selection yields smaller size for the selected gene set. On the other hand tournament selection provides slightly higher classification accuracy. We have used tournament selection in our final experiments.

Kernel Method for SVM As the kernel method for SVM, use of both linear and RBF kernels have been examined. The experimental results are presented in Table S12. The outcome exhibits better performance for linear kernel according to

both accuracy and the number of selected genes. This is because the linear kernel is suitable for high dimensional, small sample sized data [4, 5]. Also, linear kernel is reported to have shown better performance than RBF kernel for gene selection [6]. Notably however, one of the studies suggests that choice of kernel does not affect performance in most of the cases [7].

Inertia Weight (w) Update Approach For updating the inertia weight w, we have discussed two approaches (i.e., Eq. **??** and Eq. **??**). The experimental outcome for updating inertia weight is reported in Table S13. The random update method i.e., Eq. **??** gives better performance according to selected gene size while the Eq. **??** gives better accuracy. We have decided to use Eq. **??** to update the inertia weight in our final experiments.

Local Search Local search procedure is used in both employed bee and onlooker bee stages. As local search procedures, Hill Climbing (HC), Simulated Annealing (SA), and Steepest Ascent Hill Climbing with Replacement (SAHCR) are proposed for both the stages. Results employing different local search methods in these stages are reported in Table S14. For all the experiments related to local search methods we have kept the value of $probLS$ to 1.0 unless stated otherwise.

Local Search at the Employed Bee Stage Performance of Hill Climbing as the local search procedure at the employed bee stage with HC or SAHCR or SA as the local search method at the onlooker bee stage has been examined. Hill climbing shows satisfactory performance in this stage when HC or SAHCR is run at the onlooker bee stage; the result is poor when SA is run as the local search method for onlooker bee stage. Hill Climbing only exploits a potential solution. Upward movement of HC gets lost by random walk of SA at the onlooker bee stage. Hill climbing is more likely to get stuck in a local optima. So when HC is used for the employed bee stage, the possibility to get stuck in a local optima for the employed bee is higher. As a result we need combination of further exploitation and exploration by the onlooker bee when HC is applied in employed bee stage.

Implementing SA at this stage performs well only with SAHCR at the onlooker bee stage. Since SA needs parameters to be tuned to perform well, the choices of SA parameters can have a significant impact on the method's effectiveness. Here for this experiment we have used SA parameter values without tuning. So it failed to exhibit a good performance. But despite that, SAHCR at the onlooker bee stage found good solution from SA outcome of the employed bees.

Use of SAHCR at the employed bee stage gives satisfactory performance irrespective of any local search method employed at the onlooker bee stage. SAHCR tweaks multiple times in each iteration which allows it to explore enough to find a good solution. Thus application of SAHCR results in increased running time compared to SA and HC. Use of SA as the local search at the onlooker bee stage with SAHCR at the employed bee stage shows comparatively poor performance. This can be attributed to the fact that a potential solution found by SAHCR might get lost by initial random walk of SA. Thus in this stage, we need an algorithm that will help the employed bee to land in a potentially good slope so that, when the onlooker bee exploits and explores further it can achieve a good solution.

Local Search at the Onlooker Bee Stage HC at the onlooker bee stage shows poor performance. HC will provide a good solution in this stage only if it starts from a prospective slope supplied from the employed bee stage. Thus because of a lack of exploration capability, HC performs poorly at this stage.

SA at the onlooker bee stage also performs poorly. This can be attributed to the fact that SA might go downhill sometimes. Initially SA performs mostly random walk. At the onlooker bee stage, we need to upgrade the already found solution by the employed bee. Therefore, it is expected for onlooker bees to exploit more than to explore. But use of SA here might result in degradation of a potential solution. It is expected that from the employed bee stage already the individual is in the slope of a possible good solution. But because of its initial random walk, use of SA at this stage makes the probability of loosing the progress by the onlooker bee high. This mostly resulted in a poor performance.

In this stage SAHCR performs really well, because, besides exploitation it also does enough exploration to find a good solution. In fact, the algorithm performs best when SAHCR is applied in both stages. But use of SAHCR in both stages increases the running time. Performance of SAHCR at the onlooker bee stage with either HC or SA at the employed bee stage seems quite satisfactory. For onlooker bee stage both SAHCR and HC perform significantly better than SA both with respect to accuracy and selected gene size.

Hill Climbing For hill climbing the only parameter that is tuned is the iteration count when HC is applied at the employed bee stage and SAHCR is applied in the onlooker bee stage . Iteration count from 10 to 20 with step size 2 have been considered. Results of using different iteration counts for HC at the employed bee stage are presented in Table S15. The experimental results show increase of selected gene size with the increase of iteration count. Accuracy remains stable with changes in iteration count. The accuracy reached highest at the value of 14. For this experiment the probability of local search is set to 1.0.

Simulated Annealing To tune the SA parameters we applied SA at the employed bee stage and SAHCR at the onlooker bee stage. To assess the contribution of iteration count in SA, values from 10 to 20 with step size 2 have been considered. For both default (0.4) and highest (1.0) value of probability of local search the experiments are performed. The results are given in Table S16. Accuracy increase with increased iteration count and reaches its peak at the value of 14 (20) for probability of local search 1.0 (0.4). When $probLS$ is 1.0, further increase in the iteration count causes slight decrease in the accuracy. Also number of selected gene tends to decrease with higher iteration count. For both accuracy and the number of selected genes the value of 14 shows good performance which is set as the optimized value for this parameter.

For simulated annealing the parameter temperature t is tuned using different values from $\{1, 3, 5, 7, 10, 12, 15\}$. Experiments are performed for both default (0.4) and highest (1.0) value of probability of local search. Performance measure of using different values of t for SA at the onlooker bee stage is given in Table S17. Increasing the value of t causes little degradation in accuracy but number of selected genes

increases with the increase of t. Initially t is set to a high number, which causes the algorithm to random walk in the search space. So, higher value of t means that we do random walk for a longer period of time by accepting every newly created solution regardless of how good it is. As a result the local search becomes poor at exploitation, resulting in comparatively inferior solutions. In SA t decreases slowly, eventually to 0, at which point the algorithm is doing just Hill Climbing.

The rate at which we decrease t is called the algorithm's *schedule*. The parameter *schedule* of simulated annealing is tuned using different values from 0.1 to 1 with step size 0.1. Experiments are performed for both the highest (1.0) and the default (0.4) probability of local search. The results of using different values for *schedule* is presented in Table S18. The accuracy remains almost same with the change in values of *schedule*.

Steepest Ascent Hill Climbing with Replacement The values from 10 to 20 with step size 2 have been evaluated as the iteration count of SAHCR. For this experiment SAHCR is set as local search method in onlooker bee stage and SA is set as local search method in employed bee stage. The results of tuning iteration counts of SAHCR are presented in Table S19. The results show increase of accuracy with higher iteration count.

To review the consequence of applying different amount of tweak the values form $\{5, 6, 7, 8, 9, 10, 12, 15, 20\}$ are considered. The experimental outcomes are listed in Table S20. Results of this experiment show improvement of accuracy with more tweaking. The selected gene size also tends to decrease with higher number of tweaks at both the stages. For all the experiments related to different parameter of SAHCR, both the highest (1.0) and the default values of *probLS* are considered.

Percentage of Gene to be Selected from Prefiltering Step, th_n After the filtering technique is applied the genes are ranked. A percentage of top ranked genes are selected to be passed on to the next stage. The parameter th_n determines the percentage of gene to be selected. The values for tuning th_n are taken from $\{0.004, 0.01, 0.02, 0.03, 0.04, 0.045, 0.05, 0.06, 0.065, 0.07, 0.075, 0.08, 0.085, 0.09, 0.1, 0.2\}$ when Kruskal-Wallis test is utilized as the prefiltering technique. The results are presented in Table S21. We can see that too small value of th_n causes the filtering technique to discard most of the genes including the informative ones, which results in poor predictive accuracy. For this dataset, the value 0.03 (171 genes selected from the prefiltering stage) gives the highest accuracy. For higher values of th_n accuracy remains almost same. In fact the accuracy reduces a little for high values of th_n. The task of preprocessing step is to discard the irrelevant genes. So higher value of th_n gives lower accuracy because of the presence of noisy genes. Again, too lower value of th_n means informative genes are discarded in the prefiltering step. The experimental results also support the finding [8, 9, 10, 11, 12, 13] that the use of all the genes potentially hampers the classifier performance. Table S21 also reports the number of genes selected from the preprocessing stage.

Threshold to Select Gene from Prefiltering Step, th_p The p-value for each gene is computed and then all the genes are sorted according to the p-value in the filtering

step. To select the top ranked genes form Kruskal-Wallis (F-test) we need to fix a threshold and select all the genes having p-value less (greater) than the threshold. The values from $\{0.0005, 0.001, 0.002, 0.003, 0.005, 0.007, 0.009, 0.01, 0.02, 0.025, 0, 03, 0.04, 0.045\}$ are taken into consideration to tune the parameter th_p and Kruskal-Wallis was set as the prefiltering method. The results of the experiments are reported in Table S22. Increase in th_p shows rapid increase in the selected gene size. The highest accuracy is attained at the value of 0.001 (150 genes selected from prefiltering stage). For further values of th_p little declination in the accuracy is noticed. This happens because with the increase of th_p less genes are filtered which results in the selection of noisy genes for the next stage. Thus the algorithm exhibits unsatisfactory performance. From the both approaches it is apparent that for the 9_Tumors dataset informative genes are present in top 200 genes.

Weight of Individual Bee in Pheromone Deposition Equation, c_o The contribution of the current bee S_i, individual best $pbest_i$, and global best $gbest$ in pheromone laying is determined by c_0, c_1, and c_2 respectively. To find a suitable value for the parameter c_0, values from $\{0, 0.01, 0.05, 0.1, 0.2, 0.3, 0.4, 0.5, 0.6, 0.7, 0.8, 0.9\}$ are considered. The contribution of $pbest_i$ and $gbest$ is kept the same, i.e., $c_1 = c_2$. Also some of experiments for the values from $\{0.7, 0.6, 0.5, 0.4, 0.3, 0.2\}$ for the variable c_0 while keeping the contribution of $pbest_i$, i.e., c_1 to 0 is performed. The value of c_2 is calculated so that $c_0 + c_1 + c_2 = 1$. The results are presented in Table S23. From the observed results we can find that too low a value of c_0 results in low accuracy and the selected gene size. Gene subset remains small because the best solutions contribute more to the pheromone. So only small number of components receive high pheromone values and they get selected. But little exploration may cause the algorithm to get stuck at local optima. Again too high a value for c_0 masks the contribution of good solution components obtained from personal and global best solutions which also results in lower accuracy and higher selected gene size.

Maximum Number of Algorithm Iterations, MAX_ITER This parameter gives the maximum number of times the modified ABC will be run to achieve a single solution. To tune the parameter the values are taken from the range 10 to 50 with step size 10. The experimental results are presented in Table S24. From the outcome no significant correlation can be found. The accuracy remains almost the same. Change in the selected gene size can be noticed with the variation in the value of MAX_ITER, but no actual relation is found. Also from the experimental data it is observed that on average number of iterations needed by the algorithm to reach to its final result remains almost the same for various values of MAX_ITER. So we can conclude that the algorithm converges really soon. As the selected value for MAX_ITER, 20 has been chosen, because it shows the best performance. Notably, a higher value will result in a higher running time without improvement in the accuracy. Besides the value 20 shows best performance.

Number of Trials without Improvement, $limit$ A food source which cannot be improved by a predetermined number ($limit$) of trials is abandoned by its employed bee and the employed bee is converted to a scout. The variable $trial_i$ keeps track of

number of times fitness remains unchanged for the ith bee S_i . To tune the parameter $limit$, the values $\{5, 7, 10, 12, 15, 20, 25, 30, 35, 40, 100, 200, 500, 800, \infty\}$ are used. The value ∞ for the parameter $limit$ means that no food sources are abandoned by employed bees. In the other words there is no scout bees when $limit$ is set to ∞. The results are reported in Table S25. Increase in $limit$ exhibits improvement of performance according to both accuracy (Fig. S5) and the number of selected genes (Fig. S6). The accuracy is highest when the value of $limit$ is 100. From the graph presented in Fig. S5 increasing trend for accuracy is visible. But when $limit$ is very high, accuracy remains almost the same. For the number of selected gene we can see from the graph presented in Fig. S6 that when $limit$ is very low selected gene size is very high. Otherwise decreasing trend is visible. Small value of $limit$ means that the individuals get random initialization more frequently. So there is a possibility of discarding a potential solution before the individual gets chance to exhaust it amply [14]. So a good solution might be lost in the midway. Thus lower value of $limit$ results in reduction in accuracy. Increase in $limit$ also tends to increases the average number of iterations needed for the algorithm to reach in its final solution. This might be because larger value of $limit$ means less random exploration capability. So the algorithm needs more time to reach in the final solution. When $limit$ is too small or too large, the results obtained by our algorithm are worse than those produced by using the moderate values of $limit$. Therefore, results show that proper frequency of new solution production has useful effect on the solution fitness, which can perform enough explorations to improve the search ability of the algorithm. However, the balance between exploration and exploitation processes will be altered whether $limit$ is too small or too large, which will produce worse solutions and cost much more running time. The obtained accuracy is highest for the $limit$ value 100. But high value of $limit$ may result in less exploration.

Parameter Tuning of Different Evolutionary Algorithms

Different evolutionary algorithms can be considered as search method for gene selection. In this section we will present performance of gene selection for utilizing different evolutionary algorithms including genetic algorithm (GA), ACO, and ABC in this section.

Genetic Algorithm Behavior of four parameters of genetic algorithms: population size, crossover ratio (r), mutation ratio (m), and MAX_ITER are studied. The whole process is repeated for at least 15 times with genetic algorithm as search method for each parameter combination. During these runs, all the parameter values except for the parameter in consideration are set to default. The default values for the parameters are given in Table S26. The values from $\{20, 30, 40, 50\}$ are considered to study the parameter PS. The Table S27 presents the performance outcome for different values of PS. From the experimental outcome it is clearly visible that increase in population results in increased performance both according to accuracy and selected gene size upto population size 80. For further values a little performance degradation is visible. So, the value 80 is selected as optimized value for PS in GA. Values from $\{100, 200, 300, 400\}$ are considered as maximum iteration count. The value 100 is considered as default value for MAX_ITER. The

Table S28 presents the performance outcome for different values of MAX_ITER. The value 200 is selected as final value as it exhibits good performance considering both the constraints. The values from 0.6 to 1.0 with step size 0.1 are considered as crossover ratio. The default crossover rate is set to 1.0. The results are listed in Table S29. Increase in crossover ratio (r) exhibits increased performance both considering accuracy and selected gene size. But best result is found for crossover rate 0.9. For mutation ratio (m) 0.1 and 0.01 are considered for tuning. The results are presented in Table S30. Both accuracy and selected gene size decreases with high values of m. For crossover ratio the value 0.9 is considered as final value. Mutation ratio of 0.01 is set as the optimized value. For the parameter MAX_ITER the value 200 is finalized as it shows good results both according to accuracy and selected gene size.

Artificial Bee Colony For ABC, corresponding values for parameters are set according to tuning presented in Section for the proposed method. The optimized parameter settings for ABC is given in Table S31. The whole process is repeated for at least 15 times with artificial bee colony algorithm as search method. The results are presented in Table S32.

Ant Colony Optimization The whole process is repeated for at least 15 times with ant colony optimization as search method for each parameter combination. As local search method SAHCR with iteration count 12 and no. of tweaks 5 is used. The value of ρ is set to 0.8. Tuning for two parameters of ACO: population size, and MAX_ITER is done. The default values for both the parameters are set to 20. For both the parameters values from $\{20, 30, 40, 50\}$ are considered. When one of the parameters is tuned the value of other parameter is kept default. The Table S34 and S35 present the performance outcome for different values of PS and MAX_ITER respectively. For both PS and MAX_ITER best results are found for the value 40. The other parameter values are set as given in Table S33. Initially pheromone values for all the components are set to 0. While updating pheromone only the current solution and the $gbest$ is considered.

Author details
[1]AℓEDA Group, Department of CSE, BUET, Dhaka-1205 Dhaka, Bangladesh. [2]Wellcome Trust - Medical Research Council Cambridge Stem Cell Institute, University of Cambridge, Cambridge, UK.

References

1. Statnikov, A., Aliferis, C.F., Tsamardinos, I., Hardin, D., Levy, S.: A comprehensive evaluation of multicategory classification methods for microarray gene expression cancer diagnosis. Bioinformatics **21**(5), 631–643 (2005)
2. Deng, L., Ma, J., Pei, J.: Rank sum method for related gene selection and its application to tumor diagnosis. Chinese Science Bulletin **49**(15), 1652–1657 (2004)
3. Emmert-Streib, F., Dehmer, M.: Medical Biostatistics for Complex Diseases. John Wiley & Sons, ??? (2010)
4. Lan, L., Vucetic, S.: Improving accuracy of microarray classification by a simple multi-task feature selection filter. International journal of data mining and bioinformatics **5**(2), 189–208 (2011)
5. Hsu, C.-W., Chang, C.-C., Lin, C.-J., et al.: A practical guide to support vector classification (2003)
6. Debnath, R., Kurita, T.: An evolutionary approach for gene selection and classification of microarray data based on SVM error-bound theories. Biosystems **100**(1), 39–46 (2010)
7. Lee, J.W., Lee, J.B., Park, M., Song, S.H.: An extensive comparison of recent classification tools applied to microarray data. Computational Statistics & Data Analysis **48**(4), 869–885 (2005)
8. Li, W.: The-more-the-better and the-less-the-better. Bioinformatics **22**(18), 2187–2188 (2006)
9. Li, W., Yang, Y.: How many genes are needed for a discriminant microarray data analysis. In: Methods of Microarray Data Analysis, pp. 137–149. Springer, ??? (2002)
10. Stephanopoulos, G., Hwang, D., Schmitt, W.A., Misra, J., Stephanopoulos, G.: Mapping physiological states from microarray expression measurements. Bioinformatics **18**(8), 1054–1063 (2002)

11. Nguyen, D.V., Rocke, D.M.: Multi-class cancer classification via partial least squares with gene expression profiles. Bioinformatics **18**(9), 1216–1226 (2002)
12. Bicciato, S., Luchini, A., Di Bello, C.: PCA disjoint models for multiclass cancer analysis using gene expression data. Bioinformatics **19**(5), 571–578 (2003)
13. Tan, Y., Shi, L., Tong, W., Gene Hwang, G., Wang, C.: Multi-class tumor classification by discriminant partial least squares using microarray gene expression data and assessment of classification models. Computational Biology and Chemistry **28**(3), 235–243 (2004)
14. Bansal, J.C., Sharma, H., Arya, K., Deep, K., Pant, M.: Self-adaptive artificial bee colony. Optimization (ahead-of-print), 1–20 (2014)
15. Staunton, J.E., Slonim, D.K., Coller, H.A., Tamayo, P., Angelo, M.J., Park, J., Scherf, U., Lee, J.K., Reinhold, W.O., Weinstein, J.N., *et al.*: Chemosensitivity prediction by transcriptional profiling. Proceedings of the National Academy of Sciences **98**(19), 10787–10792 (2001)
16. Su, A.I., Welsh, J.B., Sapinoso, L.M., Kern, S.G., Dimitrov, P., Lapp, H., Schultz, P.G., Powell, S.M., Moskaluk, C.A., Frierson, H.F., *et al.*: Molecular classification of human carcinomas by use of gene expression signatures. Cancer research **61**(20), 7388–7393 (2001)
17. Pomeroy, S.L., Tamayo, P., Gaasenbeek, M., Sturla, L.M., Angelo, M., McLaughlin, M.E., Kim, J.Y., Goumnerova, L.C., Black, P.M., Lau, C., *et al.*: Prediction of central nervous system embryonal tumour outcome based on gene expression. Nature **415**(6870), 436–442 (2002)
18. Nutt, C.L., Mani, D., Betensky, R.A., Tamayo, P., Cairncross, J.G., Ladd, C., Pohl, U., Hartmann, C., McLaughlin, M.E., Batchelor, T.T., *et al.*: Gene expression-based classification of malignant gliomas correlates better with survival than histological classification. Cancer research **63**(7), 1602–1607 (2003)
19. Shipp, M.A., Ross, K.N., Tamayo, P., Weng, A.P., Kutok, J.L., Aguiar, R.C., Gaasenbeek, M., Angelo, M., Reich, M., Pinkus, G.S., *et al.*: Diffuse large B-cell lymphoma outcome prediction by gene-expression profiling and supervised machine learning. Nature medicine **8**(1), 68–74 (2002)
20. Golub, T.R., Slonim, D.K., Tamayo, P., Huard, C., Gaasenbeek, M., Mesirov, J.P., Coller, H., Loh, M.L., Downing, J.R., Caligiuri, M.A., *et al.*: Molecular classification of cancer: class discovery and class prediction by gene expression monitoring. science **286**(5439), 531–537 (1999)
21. Armstrong, S.A., Staunton, J.E., Silverman, L.B., Pieters, R., den Boer, M.L., Minden, M.D., Sallan, S.E., Lander, E.S., Golub, T.R., Korsmeyer, S.J.: MLL translocations specify a distinct gene expression profile that distinguishes a unique leukemia. Nature genetics **30**(1), 41–47 (2002)
22. Bhattacharjee, A., Richards, W.G., Staunton, J., Li, C., Monti, S., Vasa, P., Ladd, C., Beheshti, J., Bueno, R., Gillette, M., *et al.*: Classification of human lung carcinomas by mRNA expression profiling reveals distinct adenocarcinoma subclasses. Proceedings of the National Academy of Sciences **98**(24), 13790–13795 (2001)
23. Singh, D., Febbo, P.G., Ross, K., Jackson, D.G., Manola, J., Ladd, C., Tamayo, P., Renshaw, A.A., D'Amico, A.V., Richie, J.P., *et al.*: Gene expression correlates of clinical prostate cancer behavior. Cancer cell **1**(2), 203–209 (2002)
24. Khan, J., Wei, J.S., Ringner, M., Saal, L.H., Ladanyi, M., Westermann, F., Berthold, F., Schwab, M., Antonescu, C.R., Peterson, C., *et al.*: Classification and diagnostic prediction of cancers using gene expression profiling and artificial neural networks. Nature medicine **7**(6), 673–679 (2001)

Figures

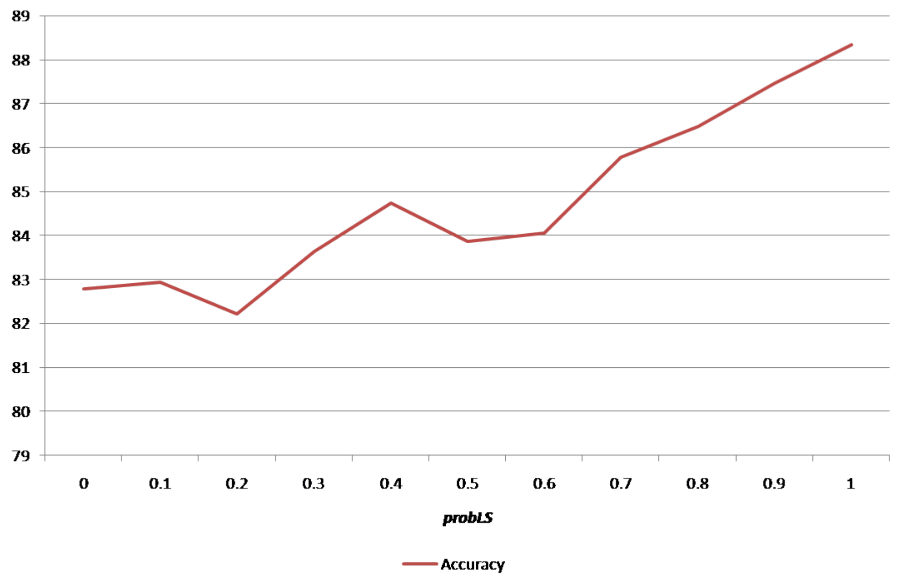

Supplementary Figure S1 Obtained accuracy with different values of $probLS$

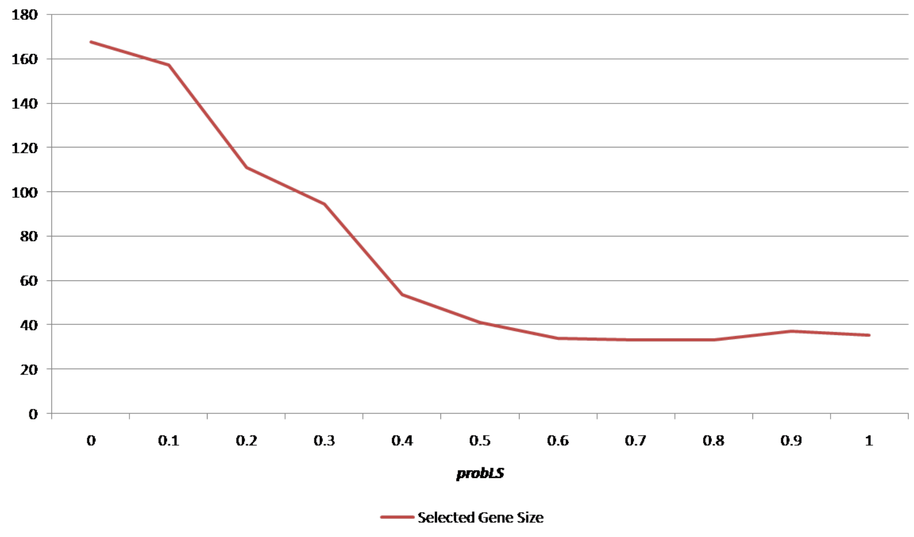

Supplementary Figure S2 Selected gene size with different values of $probLS$

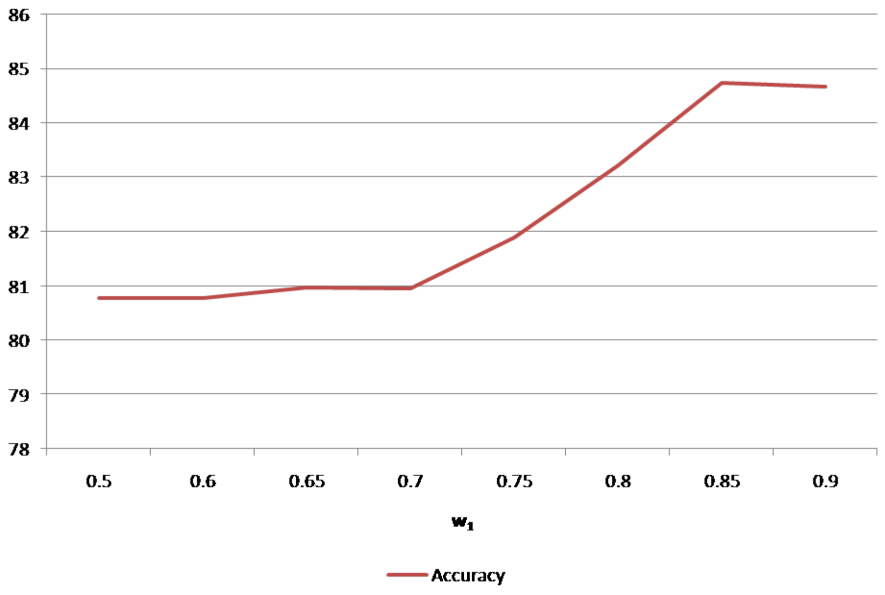

Supplementary Figure S3 Obtained accuracy with different values of w_1

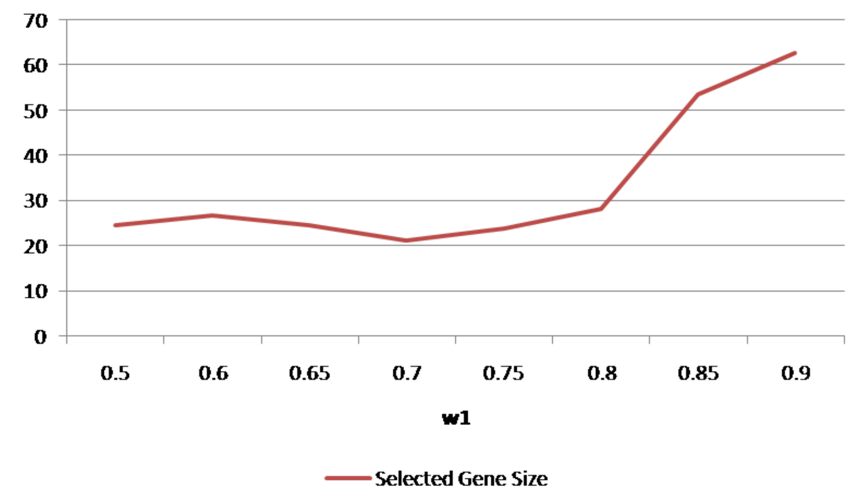

Supplementary Figure S4 Selected gene size with different values of w_1

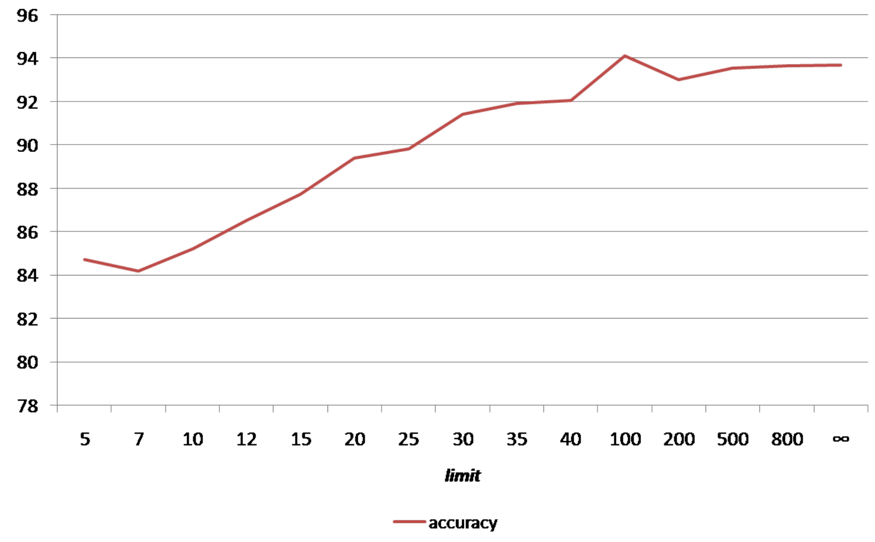

Supplementary Figure S5 Obtained accuracy with different values of $limit$

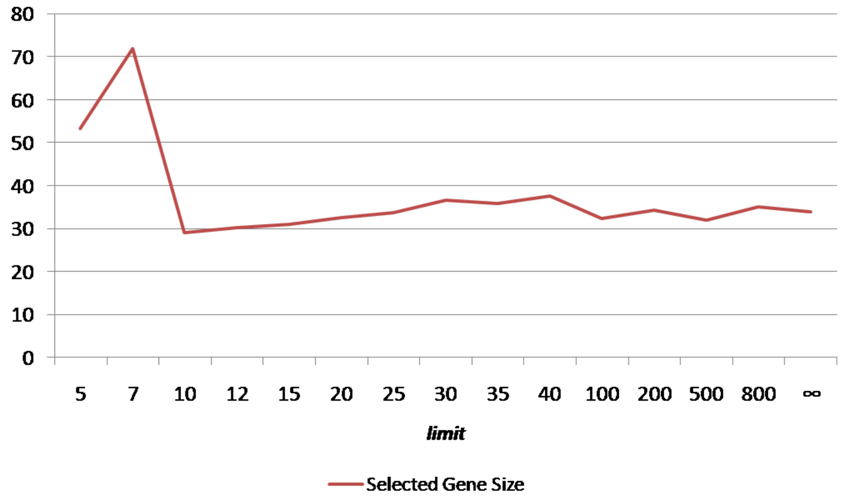

Supplementary Figure S6 Selected gene size with different values of $limit$

Tables

Supplementary Table S1 Default parameter values for tuning

Parameter	Default value	Comments
$probLS$	0.4	Probability of local search in employed and onlooker bee stage
ρ	0.8	Pheromone persistence factor
w	1.4	Inertia weight
w_1	0.85	Weight of accuracy in fitness equation
th_n	0.1	Percentage of gene to be selected from preprocessing step
hc_iter	12	Number of iterations for HC
$sahc\_iter$	12	Number of iterations for SAHCR
$sahc\_tweak$	5	Number of tweaks for SAHCR
sa_iter	12	Number of iterations for SA
t	10	Value of temperature t for SA
$schedule$	0.5	Value of $schedule$ for SA
$tmax$	5	Maximum pheromone value
$tmin$	0	Minimum pheromone value
c_0	0.5	Weight of an individual in pheromone update
MAX_ITER	20	Number of iterations
$limit$	5	Iterations to determine exhausted food source
nd	0.02	Percentage of genes to be removed in neighborhood operation
PS	20	Population size
r_4	0.1	Probability of performing $Communication\ Operation$
ls_e	SAHCR	Local search in employed bee stage
ls_o	SA	Local search in onlooker bee stage
st	Tournament Selection	Selection procedure to be used in onlooker bee stage
$kernel$	Linear	Kernel to be used in SVM
wt	Equation ??	Inertia weight update equation
uph	True	Whether to use pheromone or not
$prefilter$	Kruskal-Wallis	Prefiltering method

Supplementary Table S2 Attributes of the datasets used for experimental evaluation

Nameofthedataset	Sample size	Number of genes	Number of classes	Reference
9_Tumors	60	5,726	9	[15]
11_Tumors	174	12,533	11	[16]
Brain_Tumor1	90	5,920	5	[17]
Brain_Tumor2	50	10,367	4	[18]
DLBCL	77	5,469	2	[19]
Leukemia1	72	5,327	3	[20]
Leukemia2	72	11,225	3	[21]
Lung_Cancer	203	12,600	5	[22]
Prostate_Tumor	102	10,509	2	[23]
SRBCT	83	2,308	4	[24]

Supplementary Table S3 Performance outcome for different values of parameter r_4

Values	Accuracy		No. of selected gene	
	Avg.	S.D.	Avg.	S.D.
0	82.87	**0.02**	**25.72**	**4.3**
0.1	84.74	0.03	53.33	53.65
0.3	82.92	**0.02**	26.38	7.37
0.5	84	**0.02**	37.8	46.47
0.7	85	0.03	40.8	39.4
1	**85.52**	**0.02**	30.75	5.93

Supplementary Table S4 Performance outcome for different values of parameter uph

Values	Accuracy		No. of selected gene	
	Avg.	S.D.	Avg.	S.D.
FALSE	**85**	**0.01**	206.29	**30.15**
TRUE	84.74	0.03	**53.33**	53.65

Supplementary Table S5 Performance outcome for different values of parameter $probLS$

Values	Accuracy		No. of selected gene	
	Avg.	S.D.	Avg.	S.D.
0	82.78	0.02	167.52	45.29
0.1	82.94	0.02	157.18	67.48
0.2	82.22	0.03	111	88.85
0.3	83.63	0.03	94.35	85.1
0.4	84.74	0.03	53.33	53.65
0.5	83.86	0.03	40.95	46.77
0.6	84.06	**0.01**	33.63	6.3
0.7	85.78	0.02	**33**	9.86
0.8	86.48	0.02	33.11	**4.56**
0.9	87.46	0.02	37.05	10.05
1	**88.33**	0.02	35.13	6.28

Supplementary Table S6 Performance outcome for different values of parameter nd

Values	Accuracy		No. of selected gene	
	Avg.	S.D.	Avg.	S.D.
0.001	82.92	**0.02**	34.63	27.07
0.005	83.24	**0.02**	46.56	50.24
0.01	82.11	**0.02**	**29.07**	**7.53**
0.015	83.43	**0.02**	42.24	48.67
0.02	84.74	0.03	53.33	53.65
0.025	83.67	0.03	53.6	61.46
0.03	83.67	0.03	55.47	57.56
0.035	84.56	0.03	42.27	46.12
0.04	83.89	**0.02**	61.27	56.08
0.05	84.13	**0.02**	46.61	47.84
0.06	84.11	**0.02**	87.07	60.15
0.08	85.11	0.03	56.53	53.95
0.1	**85.22**	**0.02**	78.97	57.94
0.15	84.83	**0.02**	62.85	35.22
0.2	85.09	**0.02**	79.17	36.87

Supplementary Table S7 Performance outcome for different values of parameter ρ

Values	Accuracy		No. of selected gene	
	Avg.	S.D.	Avg.	S.D.
0	83.75	0.03	46.5	62.43
0.1	83.42	**0.02**	42.79	47.47
0.2	82.46	**0.02**	40.29	46.67
0.3	83.33	0.03	52.61	58.76
0.4	82.54	**0.02**	48.32	60.92
0.5	83.33	**0.02**	40.74	**44.62**
0.6	83.29	**0.02**	55.87	67.62
0.7	83.44	**0.02**	60.02	67.77
0.8	**84.74**	0.03	53.33	53.65
0.9	84.08	**0.02**	56.74	65.82
1	82.89	**0.02**	**37.73**	45.92

Supplementary Table S8 Performance outcome for different values of parameter w_1

Values	Accuracy		No. of selected gene	
	Avg.	S.D.	Avg.	S.D.
0.5	80.78	0.03	24.47	5.24
0.6	80.78	0.04	26.67	6.51
0.65	80.98	0.03	24.32	6.88
0.7	80.96	**0.02**	**21.05**	**2.8**
0.75	81.89	**0.02**	23.67	3.85
0.8	83.22	0.03	28.07	6.54
0.85	**84.74**	0.03	53.33	53.65
0.9	84.67	0.03	62.6	72.74

Supplementary Table S9 Performance outcome for different values of parameter PS

Values	Accuracy		No. of selected gene	
	Avg.	S.D.	Avg.	S.D.
10	82.28	**0.02**	59.52	60.21
15	83.78	0.03	73.11	73
20	84.74	0.03	53.33	53.65
25	84.83	**0.02**	53.21	56.76
30	82.98	**0.02**	**27.68**	**6.92**
35	83.75	**0.02**	38.55	43.31
40	**85.08**	**0.02**	37.1	43.38
45	84.64	0.03	34.26	31.42
50	84.39	**0.02**	33.5	30.94

Supplementary Table S10 Performance outcome for different values of parameter Prefiltering Method

Values	Accuracy		No. of selected gene	
	Avg.	S.D.	Avg.	S.D.
F-test	82.32	**0.02**	**44**	**49.72**
Kruskal-Wallis	**84.74**	0.03	53.33	53.65

Supplementary Table S11 Performance outcome for different values of parameter Selection Method

Values	Accuracy		No. of selected gene	
	Avg.	S.D.	Avg.	S.D.
Fitness-Proportionate Selection	84.2	**0.03**	**41.43**	**47.47**
Tournament Selection	**84.74**	**0.03**	53.33	53.65

Supplementary Table S12 Performance outcome for different values of parameter Kernel

Values	Accuracy		No. of selected gene	
	Avg.	S.D.	Avg.	S.D.
RBF	83.18	**0.02**	149.73	74.03
Linear	**84.74**	0.03	**53.33**	**53.65**

Supplementary Table S13 Performance outcome for different values of parameter Inertia weight update equation

Values	Accuracy		No. of selected gene	
	Avg.	S.D.	Avg.	S.D.
Eq. ??	83.78	**0.02**	**35.2**	**12.5**
Eq. ??	**84.74**	0.03	53.33	53.65

Supplementary Table S14 Performance outcome for different local search methods in employed bee and onlooker bee stage

Local search		Accuracy		No. of selected gene	
Employed Bee	Onlooker Bee	Avg.	S. D.	Avg.	S. D.
HC	HC	87.98	**0.02**	119.96	34.52
HC	SA	83.04	**0.02**	159.24	91.51
HC	SAHCR	94.91	**0.02**	58.44	16.2
SAHCR	HC	90.89	**0.02**	51.07	28.02
SAHCR	SA	88.33	**0.02**	**35.13**	**6.28**
SAHCR	SAHCR	**96.27**	**0.02**	48.06	9.41
SA	HC	85.69	0.03	53.63	36.9
SA	SAHCR	94.22	**0.02**	61.47	13.93
SA	SA	82.16	0.03	98.88	67.34

Supplementary Table S15 Performance outcome for different iteration counts for Hill Climbing in employed bee stage

Values	Accuracy		No. of selected gene	
	Avg.	S. D.	Avg.	S. D.
10	95.11	0.02	**43.73**	**6.11**
12	94.91	0.02	58.44	16.2
14	**95.19**	**0.01**	57.44	19.4
16	94.91	**0.01**	52.11	16.43
18	95.09	**0.01**	53.44	15.19
20	94.81	**0.01**	62.28	20.78

Supplementary Table S16 Performance outcome for different

iteration counts for Simulated Annealing in employed bee stage

Values	probLS 1.0				probLS 0.4			
	Accuracy		# selected gene		Accuracy		# selected gene	
	Avg.	S. D.	Avg.	S. D.	Avg.	S. D.	Avg.	S. D.
10	94.17	0.02	52	8.94	89.67	**0.02**	47.27	25.87
12	94.22	0.02	61.47	13.93	89.9	**0.02**	52.24	27.47
14	**94.9**	**0.01**	56.25	15.84	89.33	**0.02**	58.67	34.16
16	93.77	**0.01**	**49.68**	**6.38**	89.38	**0.02**	56.38	32.85
18	93.98	0.02	55.94	16.59	89	**0.02**	60.65	36.69
20	93.93	0.02	58.64	19.54	**90.48**	0.03	**45.04**	**17.07**

Supplementary Table S17 Performance outcome for different temperature (t) values for Simulated Annealing in employed bee stage

Values	probLS 1.0				probLS 0.4			
	Accuracy		# selected gene		Accuracy		# selected gene	
	Avg.	S. D.	Avg.	S. D.	Avg.	S. D.	Avg.	S. D.
1	94.54	0.02	55.28	17.35	89.44	**0.02**	56.33	33.72
3	**95**	**0.01**	53.53	14.88	88.26	0.03	73.91	50.12
5	94.12	0.02	55.06	20.4	89.24	0.03	52.5	28.85
7	94.11	**0.01**	56.93	17.39	89.21	0.03	53.05	24.6
10	94.22	0.02	61.47	13.93	**89.9**	**0.02**	52.24	27.47
12	93.82	0.02	**52**	**10.91**	89	**0.02**	52.47	19.18
15	94.38	0.02	67.38	22.01	89.27	**0.02**	**44**	**9.47**

Supplementary Table S18 Performance outcome for different values of the parameter $schedule$ for Simulated Annealing in employed bee stage

Values	probLS 1.0				probLS 0.4			
	Accuracy		# selected gene		Accuracy		# selected gene	
	Avg.	S. D.	Avg.	S. D.	Avg.	S. D.	Avg.	S. D.
0.1	93.51	0.02	58.74	15.82	**90.56**	**0.02**	**44.13**	**7.07**
0.2	94.22	0.02	52.53	16	89.78	**0.02**	52.73	20.97
0.3	93.89	0.02	56.47	10.9	89.74	**0.02**	59.63	24.61
0.4	94.22	0.02	51.2	18.04	89.67	**0.02**	53.52	23.17
0.5	94.22	0.02	61.47	13.93	89.9	**0.02**	52.24	27.47
0.6	94.33	**0.01**	54.07	17.02	88.67	0.03	46.07	26.73
0.7	93.56	**0.01**	53.87	**9.32**	89.56	0.03	49.67	24.76
0.8	93.86	**0.01**	**50.11**	12.13	90.22	0.03	52.2	29.81
0.9	**94.6**	0.02	54.9	15.59	89.22	0.03	54.47	39.27
1	94.48	0.02	51.94	14.22	89	0.03	60.2	35.32

Supplementary Table S19 Performance outcome for different iteration counts for Steepest Ascent Hill Climbing with Replacement in onlooker bee stage

Values	probLS 1.0				probLS 0.4			
	Accuracy		# selected gene		Accuracy		# selected gene	
	Avg.	S. D.	Avg.	S. D.	Avg.	S. D.	Avg.	S. D.
10	94.13	0.02	**49.05**	**7.95**	88.61	**0.02**	**43.89**	20.55
12	94.22	0.02	61.47	13.93	89.9	**0.02**	52.24	27.47
14	95.33	0.02	61.8	15.47	90.67	0.03	54.27	22.08
16	95.42	0.02	56.13	13.9	90.94	**0.02**	44.13	**9.13**
18	95.59	**0.01**	65.88	15.09	91.83	**0.02**	58.43	29.58
20	**96.56**	**0.01**	65.63	10.68	**92.11**	**0.02**	60.67	15.51

Supplementary Table S20 Performance outcome for different values of the parameter $tweak$ for Steepest Ascent Hill Climbing with Replacement

Values	probLS 1.0				probLS 0.4			
	Accuracy		# selected gene		Accuracy		# selected gene	
	Avg.	S. D.	Avg.	S. D.	Avg.	S. D.	Avg.	S. D.
5	94.22	0.02	61.47	13.93	89.9	**0.02**	52.24	27.47
6	95.37	0.02	50.94	10.41	89.39	**0.02**	45.18	25.57
7	94.8	0.02	50.29	16.29	89.78	**0.02**	47	11
8	95.56	0.02	47.6	13.06	91.59	0.03	40.41	**5.66**
9	96.33	**0.01**	48.75	12.11	91.62	0.03	43.61	16.07
10	96.14	0.02	48.32	9.97	92.55	**0.02**	46.18	19.63
12	96.85	**0.01**	52.67	14.65	93.13	0.03	48.06	14.84
15	96.97	**0.01**	46.41	10.98	92.78	**0.02**	**36.2**	6.21
20	**98.04**	**0.01**	**41.76**	**6.33**	**93.52**	**0.02**	41.22	14.74

Supplementary Table S21 Performance outcome for percentage of genes selected in prefiltering stage, th_n

Values	# genes selected from prefiltering stage	Accuracy		No. of selected gene	
		Avg.	S.D.	Avg.	S.D.
0.004	22	73.78	0.02	**9.2**	**1.9**
0.01	57	83	**0.01**	21.33	2.82
0.02	114	86.11	**0.01**	34.4	9.16
0.03	171	**86.81**	0.02	41.43	20.35
0.04	229	85.45	0.02	41.36	24.47
0.045	257	85.25	0.03	47.11	30.02
0.05	286	85	0.03	47.27	34.65
0.06	343	83.11	0.02	30.87	24.66
0.065	372	85.33	0.03	51	45.36
0.07	400	84.58	0.02	36	32.17
0.075	429	84.21	0.03	33.58	24.98
0.08	458	85.33	0.02	55.27	51.97
0.085	486	84.56	0.02	50.47	56.56
0.09	515	83.67	0.02	48.6	51.35
0.1	572	84.74	0.03	53.33	53.65
0.2	1145	84.38	0.02	171.63	193.37

Supplementary Table S22 Performance outcome for threshold of p-value in prefiltering stage, th_p

Values	# genes selected from prefiltering stage	Accuracy		No. of selected gene	
		Avg.	S.D.	Avg.	S.D.
0.0005	91	85.89	0.02	**27.53**	**7.33**
0.001	150	**85.92**	**0.01**	36.9	12.69
0.002	235	85.21	0.02	43.58	25.61
0.003	298	85.1	0.03	39.5	30.17
0.005	413	84.82	0.02	64.05	51.94
0.007	506	84.56	0.02	56.47	52.2
0.009	577	83.73	0.02	48.05	54.48
0.01	605	83.52	0.03	73.5	78.97
0.02	867	83.86	0.02	91.32	117.88
0.025	989	84.44	0.02	145.39	147.8
0.03	1083	83.92	0.02	139.1	167.76
0.04	1259	85.1	0.02	234.75	213.27
0.045	1327	84.31	0.03	196.94	202.61

Supplementary Table S23 Performance outcome for different values of c_0

c_0	c_1	c_2	Accuracy		No. of selected gene	
			Average	S. D.	Average	S. D.
0	0.5	0.5	83.75	0.02	34.8	28.57
0.01	0.495	0.495	83	0.02	29.87	8
0.05	0.475	0.475	83.22	0.02	62.6	64.16
0.1	0.45	0.45	83.25	0.02	60.32	65.14
0.2	0.4	0.4	82.4	0.02	41.56	52.24
0.3	0.35	0.35	83.22	0.03	61.13	70.85
0.4	0.3	0.3	83.44	0.03	79.13	82.08
0.5	0.25	0.25	**84.74**	0.03	53.33	53.65
0.6	0.2	0.2	84.35	0.04	37.94	38.5
0.7	0.15	0.15	83.79	0.03	69.82	72.87
0.8	0.1	0.1	82.78	0.02	42.07	49.95
0.9	0.05	0.05	83.67	0.03	58.8	71.09
0.2	0	0.8	84.69	0.03	58	65.48
0.3	0	0.7	84	0.04	**27.87**	**6.15**
0.4	0	0.6	83.13	0.03	29.88	8.35
0.5	0	0.5	82.65	**0.01**	38.53	49.01
0.6	0	0.4	83.11	0.02	35.73	39.98
0.7	0	0.3	84	0.02	42	55.26

Supplementary Table S24 Performance outcome for different values of parameter MAX_ITER

Values	Accuracy		No. of selected gene		Average number of iterations needed to reach the final solution
	Avg.	S.D.	Avg.	S.D.	
10	83.78	0.03	44.2	**48.33**	5.07
20	**84.74**	0.03	53.33	53.65	6.27
30	83.92	0.03	46.12	59.66	5.47
40	84.22	**0.02**	**40.6**	44.42	6.6
50	83.44	**0.02**	51.67	61.27	7.93

Supplementary Table S25 Performance outcome for different values of parameter $limit$

**Values**	**Accuracy**		**No. of selected gene**		Average number of iterations
	Avg.	S.D.	Avg.	S.D.	needed to reach the final solution
5	84.74	0.03	53.33	53.65	6.27
7	84.21	0.03	71.95	66.13	7.37
10	85.22	**0.02**	**29.13**	7.03	7.13
12	86.56	**0.02**	30.27	6.63	6.93
15	87.75	**0.02**	31.15	**5.22**	7.75
20	89.42	**0.02**	32.65	6.17	9.3
25	89.82	**0.02**	33.79	8.22	11
30	91.42	**0.02**	36.75	6.64	11.85
35	91.94	**0.02**	35.89	5.85	11.61
40	92.08	**0.02**	37.69	7.36	15.94
100	**94.12**	**0.02**	32.35	5.96	18.53
200	93.04	**0.02**	34.44	7.97	17.94
500	93.56	**0.02**	31.97	6.85	18.67
800	93.66	**0.02**	35.08	7.1	18.75
∞	93.7	**0.02**	33.89	8.28	19.26

Supplementary Table S26 Default values for tuning GA parameters

Parameter	Default value
PS	20
MAX_ITER	100
r	1.0
m	0.1

Supplementary Table S27 Performance outcome for different values of PS for GA

Values	Accuracy		No. of selected gene	
	Avg.	S. D.	Avg.	S. D.
20	80.48	**0.01**	281.07	11.21
30	80.89	**0.01**	279.97	9.93
40	80.97	**0.01**	278.67	12.37
50	81.44	**0.01**	278.8	12.95
60	81.56	**0.01**	**276.13**	**7.06**
70	81.67	**0.01**	279.07	11.98
80	**81.78**	**0.01**	280.6	11.38
90	81.44	**0.01**	276.8	11.91
100	81.67	**0.01**	277.47	15.38

Supplementary Table S28 Performance outcome for different values of MAX_ITER for GA

Values	Accuracy		No. of selected gene	
	Avg.	S. D.	Avg.	S. D.
100	80.48	**0.01**	281.07	11.21
200	80.72	**0.01**	282.3	10.31
300	80.33	**0.01**	**277.6**	**9.86**
400	**80.89**	**0.01**	285.8	15.06

Supplementary Table S29 Performance outcome for different values of r for GA

Values	Accuracy		No. of selected gene	
	Avg.	S. D.	Avg.	S. D.
1	80.48	**0.01**	281.07	**11.21**
0.9	**80.83**	**0.01**	**280.93**	12
0.8	80.44	**0.01**	281.97	11.94
0.7	79.78	**0.01**	283.7	14.4
0.6	80.17	**0.01**	284.57	14.12

Supplementary Table S30 Performance outcome for different values of m for GA

Values	Accuracy		No. of selected gene	
	Avg.	S. D.	Avg.	S. D.
0.01	**80.48**	**0.01**	281.07	11.21
0.1	79.94	**0.01**	**277.83**	**10.61**

Supplementary Table S31 Parameter values for tuning ABC parameters

Parameter	Value
w_1	0.85
th_n	0.065
MAX_ITER	20
$limit$	35
nd	0.035
PS	25
selection method	Tournament Selection
$kernel$	Linear
$prefilter$	Kruskal-Wallis

Supplementary Table S32 Performance of Artificial Bee Colony algorithm in gene selection

Criteria	Best	Avg.	S.D.	Worst
Accuracy	91.67	87.67	0.02	85
# Genes	57	75.67	14.18	97

Supplementary Table S33 Default values for tuning ACO parameters

Parameter	Default value	Comments
ρ	0.8	Pheromone persistence factor
w	1.4	Inertia weight
w_1	0.85	Weight of accuracy in fitness equation
th_n	0.1	Percentage of gene to be selected from preprocessing step
$sahc\_iter$	12	Number of iterations for SAHCR
$sahc\_tweak$	5	Number of tweaks for SAHCR
$tmax$	5	Maximum pheromone value
$tmin$	0	Minimum pheromone value
c_0	0.5	Weight of an individual in pheromone update
MAX_ITER	40	Number of iterations
nd	0.02	Percentage of genes to be removed in neighborhood operation
PS	40	Population size
$kernel$	Linear	Kernel to be used in SVM
wt	Equation ??	Inertia weight update equation
uph	True	Whether to use pheromone or not
$prefilter$	Kruskal-Wallis	Prefiltering method

Supplementary Table S34 Performance outcome for different values of PS for ACO

Values	Accuracy		No. of selected gene	
	Avg.	S. D.	Avg.	S. D.
20	81.83	**0.01**	275.03	14.93
30	82.78	**0.01**	272.89	**11**
40	**82.93**	**0.01**	**271.24**	11.95
50	82.78	**0.01**	273.33	12.97

Supplementary Table S35 Performance outcome for different values of MAX_ITER for ACO

Values	Accuracy		No. of selected gene	
	Avg.	S. D.	Avg.	S. D.
20	81.83	**0.01**	275.03	14.93
30	81.67	**0.01**	**270.24**	13.87
40	**82.16**	**0.01**	273.41	**9.67**
50	81.48	0.02	270.48	13.02
